# Poly(ADP-ribosyl)ating enzymes coordinate changes in the expression of metabolic genes with developmental progression

**DOI:** 10.1038/s41598-023-47691-8

**Published:** 2023-11-20

**Authors:** Guillaume Bordet, Gbolahan Bamgbose, Alexei V. Tulin

**Affiliations:** Department of Biomedical Sciences, School of Medicine and Health Sciences, University of North Dakota, 501 North Columbia Road, Stop 9061, Grand Forks, ND 58202 USA

**Keywords:** Developmental biology, Genetics

## Abstract

Metabolism, known to be temporally regulated to meet evolving energy demands, plays a crucial role in shaping developmental pace. Recent studies have demonstrated that two key proteins PARP1 and PARG play a regulatory role in the transcription of both morphogenic and metabolic genes. Intriguingly, in *Drosophila*, the depletion of PARP1 or PARG proteins causes a developmental arrest before pupation, resulting in individuals unable to complete their development. This phenotype highlights the critical involvement of poly(ADP-ribosyl)ating enzymes in regulating the metamorphic process. In this study, we provide compelling evidence that these enzymes intricately coordinate transcriptional changes in both developmental and metabolic pathways during metamorphosis. Specifically, they promote the expression of genes crucial for pupation, while simultaneously negatively regulating the expression of metabolic genes before the transition to the pupal stage. Additionally, these enzymes suppress the expression of genes that are no longer required during this transformative period. Our findings shed light on the intricate interplay between poly(ADP-ribosyl)ating enzymes, developmental processes, and metabolic regulation before metamorphosis and highlight a new role of poly(ADP-ribosyl)ating enzymes in the global regulation of transcription.

## Introduction

The orchestration of multicellular organism development relies on the sequential progression of proliferation and differentiation, each phase being accompanied by the activation of specific gene expression programs and diverse energy requirements^1,2^. Previous studies have demonstrated that metabolism is not a constant process throughout an organism's life but rather exhibits temporal regulation to match the dynamic energy demands^3,4^. Additionally, it has been observed that metabolism influences the pace of development^5^, further emphasizing the need for coordinated regulation between developmental progression and metabolic activities.

Poly(ADP-ribose) polymerase 1 (PARP1), a chromatin-associated protein, has emerged as a critical player in development^6,7^. PARP1 synthesizes poly(ADP-ribose) chains that modify chromatin proteins, resulting in their dissociation from DNA and subsequent chromatin opening^8–10^. Conversely, poly(ADP-ribose) glycohydrolase (PARG) reverses PARP1 activity by removing poly(ADP-ribose) moieties from acceptor proteins^11–13^. Recent studies have revealed a complementary, rather than antagonistic, relationship between PARG and PARP1^12,14–16^. Upon activation, PARP1 undergoes automodification and dissociates from chromatin, leading to its inactivation^8,17,18^. In contrast, by removing poly(ADP-ribose) moieties from PARP1, PARG allows for its re-association with chromatin.

In fruit flies, both *parp1*^*C03256*^ hypomorph and *parg*^*27.1*^ null mutant animals suffer developmental arrest just prior to pupation stage, while their embryonic and larval development is temporally comparable to wildtype animals^11,18^. Consequently, the animals are viable but remain third instar larvae, unable to complete their development^7^. Previous findings suggest that both PARG and PARP1 regulate the transcription of developmental genes during the larva-to-pupa transition^14^. However, PARG lacks a DNA-binding domain (DBD) and seems to interact with chromatin only to a limited extent^19^, the specific interaction between PARG and chromatin in the context of the protein’s recruitment remains to be elucidated. We hypothesized that PARG is recruited to regulate transcription through its interaction with PARP1. To investigate this possibility, we conducted chromatin immunoprecipitation (ChIP) of PARG and PARP1 during the late third instar larval stage. Our results demonstrate that both PARP1 and PARG directly control global changes in the transcriptional profile that occurs prior to metamorphosis for both metabolic and developmental processes but through distinct mechanisms. Firstly, together with PARP1, PARG binds to the promoter region of genes crucial for pupation, promoting their expression. Secondly, in the absence of PARP1, PARG binds to the gene body of metabolic genes, repressing their expression, and binds to genes that are no longer required at the end of larval stages, repressing their expression as well. Together, these results suggest that PARP1 and PARG coordinate the regulation of metabolism and developmental genes during developmental progression.

## Results

### PARG binds to chromatin.

To assess the chromatin-binding capacity of PARG, we conducted chromatin immunoprecipitation followed by sequencing (ChIP-seq) assays using wandering third instar larvae (puffstage 7–9), expressing PARG-YFP in an endogenous *parg* mutant background, which was compared to a yellow white control line lacking GFP expression. We specifically chose this stage of wandering third instar larvae because it is the final stage before *parg* and *parp1* mutants experience developmental arrest. Furthermore, the timing of this developmental stage is sharp, limiting potential developmental disparities between animals^20^. We have previously shown that PARG-YFP effectively rescues the developmental arrest observed in *parg*^*27.1*^ null mutants^13^ and exhibits, on average, a 60% higher expression level compared to endogenous Parg (Supplemental Fig. S1). However, this difference is not statistically significant when considering the inherent variability within the populations. Furthermore, PARG-YFP does not display any discernible phenotypic changes^21^, suggesting that PARG-YFP functions similarly to endogenous PARG.

**Figure 1:**
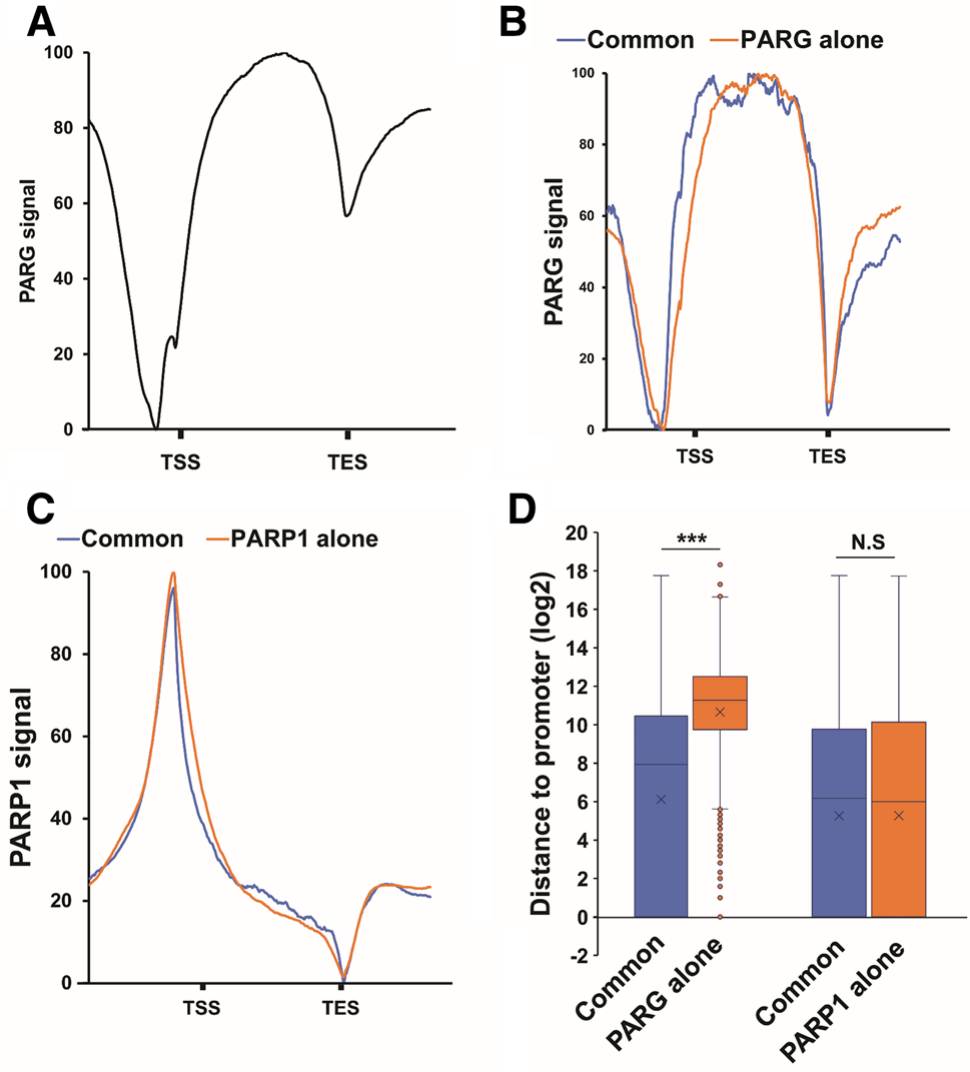
PARG binds to chromatin. (**A**) Graph representing the average distribution of PARG along the genes. On the X-axis, the region of all target genes is scaled to a 2 kb region (from TSS to TES) and includes the 1 kb region upstream from TSS and downstream from TES. On the Y-axis, the PARG signal is normalized to the lower (0%) and the higher value (100%) to better visualize the difference in distribution. This graph was generated using all the genes bound by PARG (PARG and PARG/PARP1 categories of Supplemental Table S1). (**B**) Graph representing the average distribution of PARG along the PARG/PARP1 common target genes (blue) or PARG-alone target genes (orange) (respectively PARG/PARP1 and PARG categories of Supplemental Table S1). (**C**) Graph representing the average distribution of PARP1 along the PARG/PARP1 common target genes (blue) or PARP1- alone target genes (orange) (respectively PARG/PARP1 and PARP1 categories of Supplemental Table S1). (**D**) Distribution of the distance of PARG (left) and PARP1 (right) peaks to promoter in log2 scales for the promoter of PARG/PARP1 common targets (blue) and PARG- or PARP1-alone targets (orange) (respectively PARG/PARP1 and PARG or PARP1 categories of Supplemental Table S1). The significance of the difference between the two populations was addressed with a Mann–Whitney U test. ***: p-value < 0.01. N.S: Non-significant.

We identified that PARG binds to 4639 genes (Supplemental Table S1). PARG is mainly enriched in the gene body and is almost depleted to the promoter region, except at 30 bp, upstream of the transcription start site (TSS) (Fig. 1A) (Supplemental Fig. S2). To confirm PARG binding, we performed ChIP-qPCR on *kek1* locus, one of the top peaks (Supplemental Fig. S3). Since PARG protein does not have a DNA-binding domain (DBD), our central hypothesis was that PARG is recruited to chromatin via PARP1. Accordingly, we expected PARG and PARP1 chromatin binding profiles to be similar. Interestingly, however, we found that only 57.1% (2649) of PARG binding loci are shared with PARP1 (Supplemental Fig. S4) (Supplemental Table S1). Moreover, we observed that PARG is more enriched to the promoter region of PARG/PARP1 common targets compared to PARG-alone targets (Fig. 1B). In contrast, PARP1 distribution is similar between common and PARP1- alone targets (Fig. 1C). In parallel, we analyzed the distance between PARG peaks and the promoter. Results revealed that PARG binds significantly closer to the promoter of common targets, while PARP1 peaks show no significant differences (Fig. 1D). Collectively, these results show that PARG binds to chromatin and that its binding profile is affected by the presence of PARP1.

### PARG presents two distinct binding profiles

**Figure 2:**
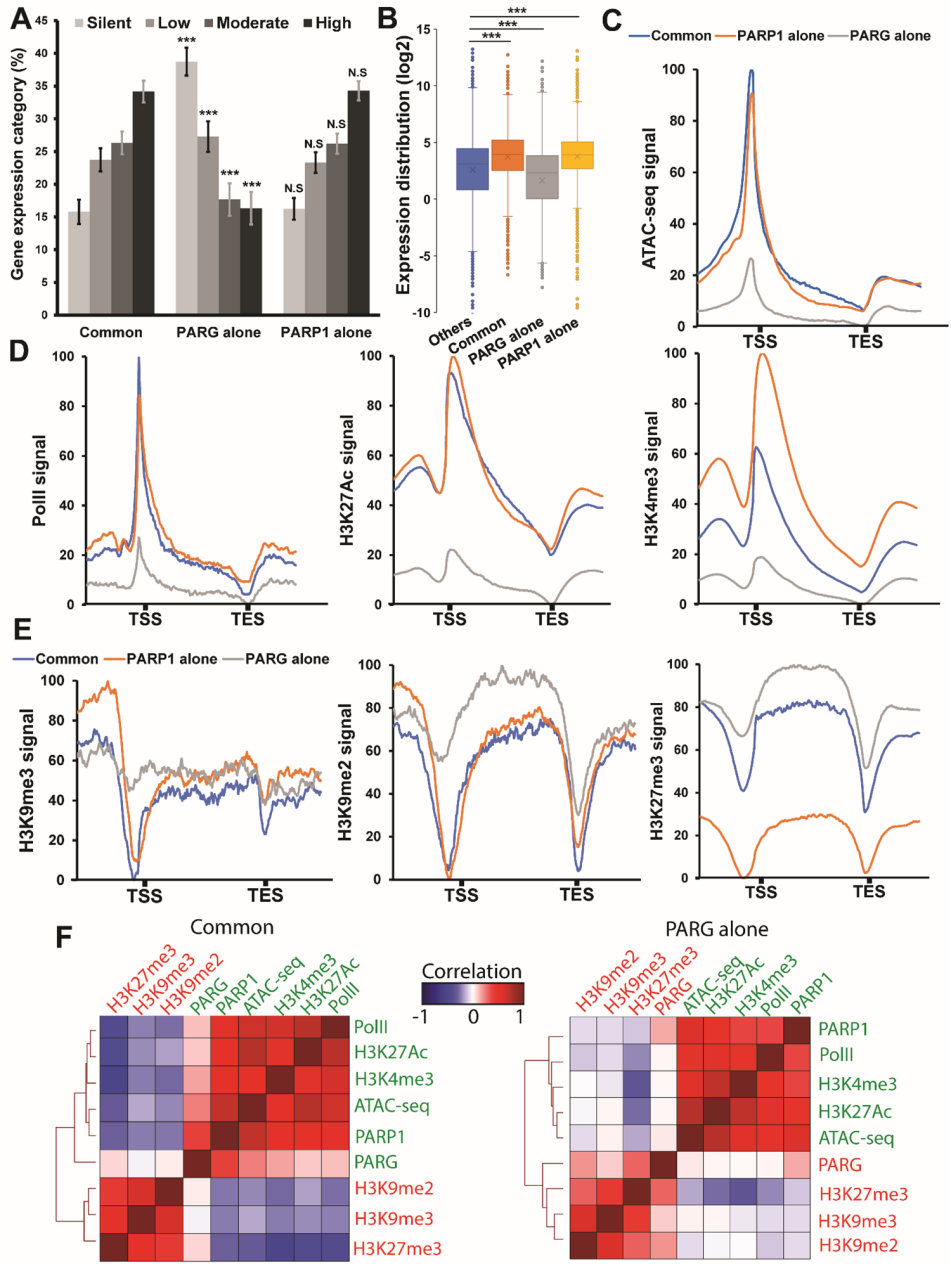
The presence of PARP1 affects PARG-binding profile. (**A**) Expression level of PARG-alone, PARP1-alone, and PARG/PARP1 common target genes. Genes were sorted based on their expression level during late third instar larvae (larval puffstage 7-9)^23^ and split into four quartiles, each including 25% of the genes. From the lowest to the highest expression, the quartiles were named as follows: Silent, Low, Moderate and High. The Y-axis represents the number of genes in percentage that belong to each group for PARG/PARP1 common targets (left), PARG-alone (middle), and PARP1-alone targets (right) (respectively PARG/PARP1, PARG and PARP1 categories of Supplemental Table S1). Error bars are standard error of the proportion and statistical test was Fisher exact test, PARG alone and PARP1 alone were compared to Common. ***: p-value < 0.01, N.S.: Non-significant. (**B**) Box plot representing the distribution of the log2 expression of PARG/PARP1 common targets (orange), PARG-alone (gray), PARP1- alone (yellow), and all other gene targets (gray) during late third instar larvae^23^. The difference among populations was analyzed with a Mann–Whitney U test. ***: p-value < 0.01. (**C**) Third instar larvae ATAC-seq signal^22^ to quantify chromatin accessibility for PARG/PARP1 common targets (blue), PARP1-alone (orange) and PARG-alone targets (gray). (**D**) Occupancy of polymerase II (Pol II) (left) and occupancy of the active marks H3K27Ac (middle) and H3K4me3 (right) to PARG/PARP1 common targets (blue), PARP1-alone (orange), or PARG-alone targets (gray). (**E**) Occupancy of the repressive marks H3K9me3 (left), H3K9me2 (middle), and H3K27me3 (right) to PARG/PARP1 common targets (blue) or PARP1-alone (orange), or PARG-alone targets (gray). (**F**) Spearman correlation of genome-wide signal for PARG/PARP1 common targets (left) or PARG-alone (right) targets (respectively PARG/PARP1 and PARG categories of Supplemental Table S1). (**D**, **E**) See the “Methods” section for information about the datasets that were used.

Next, we investigated the transcription profile of PARG and PARP1 targets during late third instar larvae. We found that PARG/PARP1 common and PARP1-alone targets are mainly highly expressed genes, while PARG-alone targets are mainly silent genes (Fig. 2A). Furthermore, the expression of PARG-alone targets is, on average, significantly lower, while PARG/PARP1 common and PARP1-alone target expression is higher compared to other genes (Fig. 2B). Then, we analyzed the chromatin accessibility of PARP1 and PARG targets using the publicly available third instar larvae Assay for Transposase-Accessible Chromatin (ATAC) data^22^. This analysis revealed that PARG/PARP1 common and PARP1-alone target loci are more accessible than PARG- alone target loci (Fig. 2C). Moreover, the occupancy of active chromatin marks (H3K4me3 and H3K27Ac), but also PolII occupancy, is higher in PARG/PARP1 common and PARP1-alone targets while the distribution pattern of these markers is not affected (Fig. 2D). Specifically, at the promoters of common and PARP1-alone targets, the occupancy of PolII and H3K27Ac was found to be five times higher than that observed at PARG-alone target promoters. Similarly, at the promoters of PARP1-alone targets and common targets, H3K4me3 occupancy was five and three times higher, respectively, than at PARG-alone target promoters. In contrast, the occupancy of repressive chromatin marks (H3K27me3, H3K9me3 and H3K9me2) is higher for PARG-alone targets while the distribution pattern of these markers is not affected (Fig. 2E). Specifically, H3K9me3 was observed at the promoters of PARG-alone targets but absent at the promoters of common and PARP1-alone targets. Moreover, the gene bodies of PARG-alone targets exhibited a 43% increase in H3K9me2 occupancy compared to common and PARP1-alone targets. Furthermore, H3K27me3 occupancy was found to be five times higher at the gene bodies of PARG-alone targets and four times higher at the gene bodies of common targets compared to PARP1-alone targets. Overall, PARG binding profile correlates more with active marks for common targets (Fig. 2F), while it correlates more with repressive marks for PARG-alone targets (Fig. 2F). Taken together, these results suggest that PARG binds to highly active genes when PARP1 is present, and to silent genes when alone.

### PARG regulates the expression of its target genes

**Figure 3:**
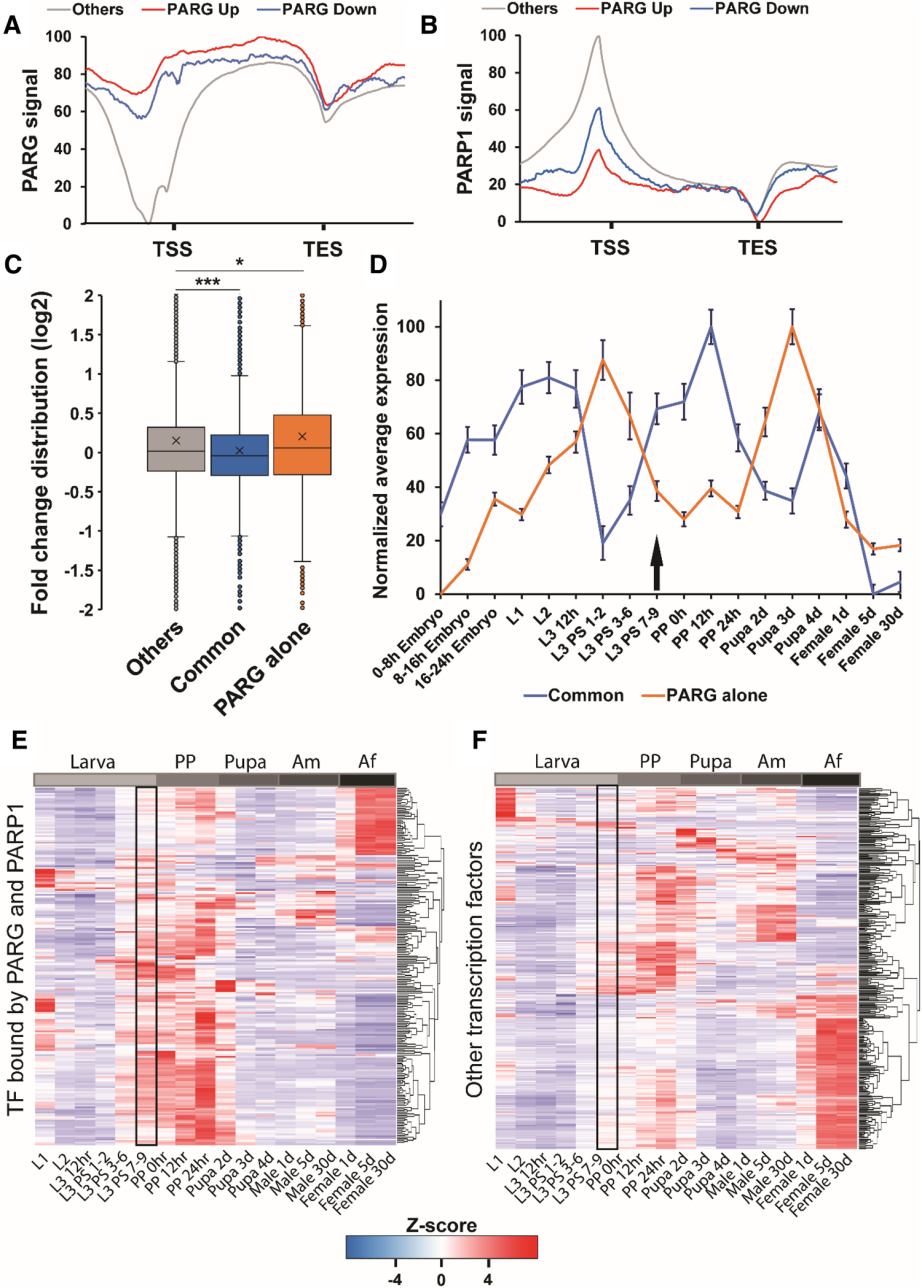
PARG regulates the expression of its target genes. (**A,** **B**) Average distribution of PARG (**A**) or PARP1 (**B**) along the genes upregulated (red), downregulated (blue) or those not misregulated (gray) in *parg* mutant. On the X-axis, the region of all target genes is scaled to a 2 kb region (from TSS to TES) and includes the 1 kb region upstream from TSS and downstream from TES. On the Y-axis, the distribution of PARG is normalized to the lower (0%) and the higher value (100%) to better visualize the difference in distribution. (**C**) Box plot representing the distribution of the log2 fold change of PARG/PARP1 common targets (blue), PARG-alone (orange), and all other gene targets (gray) during late third instar larvae in *parg* mutant compared to wild-type larvae. Difference among populations was analyzed with a with a Mann–Whitney U test. *: p-value < 0.05. ***: p-value < 0.01. (**D**) Average time course expression of PARG/PARP1 common (blue) and PARG-alone (orange) target genes from embryo to adult. Average expression is normalized from 0% (lowest expression) to 100% (highest expression). Error bar is standard error of the mean (SEM). Black arrow indicates the studied stage. L1, First instar larvae; L2, Second instar larvae; L3, Third instar larvae; h, Hours; PS, puff stage; PP, Prepupa; d, Days. Time course expression data were obtained from^23^. (**E**, **F**) Heatmap representing the time course expression of genes coding for a transcription factor bound by both PARG and PARP1 (**E**) and all other genes coding for a transcription factor (**F**) from first instar larvae to 30-day-old adult flies. Normalized expressions are shown as row z-scores. L1, First instar larvae; L2, Second instar larvae; L3, Third instar larvae; hr, Hours; PS, puff stage; PP, Prepupa; d, Days; Am, Adult males; Af, Adult females. Time course expression data were obtained from^23^.

We previously reported that PARG controls the transcription of developmental genes during third instar larvae, mainly as a repressor of transcription^14^. We then investigated if the expression of PARG/PARP1 common and PARG-alone targets is affected differently in the absence of PARG. First, we found that PARG binds to the loci of 68.3% of the genes significantly misregulated in *parg* mutant. Conversely, only 62.8% of the genes bound by PARG are misregulated in *parg* mutant. However, PARG presents a higher occupancy to the loci of genes that are significantly misregulated in *parg* mutant compared to other genes (Fig. 3A). Interestingly, PARP1 presents a higher occupancy to genes significantly downregulated in the absence of PARG (Fig. 3B). Furthermore, PARG/PARP1 common targets are significantly downregulated in *parg* mutant, while PARG-alone targets are significantly upregulated (Fig. 3C). Surprisingly, 72.1% of PARG-alone targets are also upregulated in *parp1* mutant while PARP1 does not bind to them. This suggests that PARG promotes gene expression when PARP1 is present and participates in repression of expression when alone.

### PARG/PARP1 common targets are developmental genes upregulated during pupal stage

Next, we analyzed the time course expression of PARG/PARP1 common target genes in wild-type animals^23^. Overall, PARG/PARP1 common targets are genes that present a high expression during late instar larval stages, with a peak of expression during pupal stage (Fig. 3D, Supplemental Fig. S5A) with around 60% of PARG/PARP1 common targets upregulated during the transition from early to late third instar larvae (Supplemental Fig. 5B). In contrast, PARP1-alone target genes present high expression along all larval stages (Supplemental Fig. 5C). Gene ontology analysis reveals that PARG/PARP1 common target genes are involved in development, morphogenesis, cell differentiation and metamorphosis (Supplemental Fig. S6). A specific subset of 134 genes among the PARG/PARP1 common targets are specifically implicated in the process of metamorphosis (Supplemental Table S2). These genes exhibit a notable increase in expression prior to the pupal transition while their expression is reduced in both *parg* and *parp1* mutants (Supplemental Table S2). Furthermore, among PARG/PARP1 common targets, we identified 196 genes coding for transcription factors (Supplemental Table S3). These transcription factors are mainly expressed during the end of third instar larvae and pupal stage (Fig. 3E, F, Supplemental Fig. 5D). Among them, we identified several transcription factors involved in ecdysone-response, including *br*, *eip74ef*, *eip75b*, *eip78c*, *hr3*, *hr38*, and *ftz-f1*, but also the gene coding for ecdysone receptor (*ecr*) that plays a critical role during metamorphosis^24,25^. Collectively, our findings underscore the collaborative role of PARG and PARP1 in promoting the expression of critical developmental genes that play a pivotal role in metamorphosis.

### PARG binds in the absence of PARP1 to the loci of metabolic genes and of cuticle components that are downregulated before pupal stage

Finally, we analyzed the time course expression of PARG-alone target genes in wild-type animals^23^. Overall, PARG-alone targets are genes silenced during late third instar larvae and start their expression again during late pupa (Fig. 3D, Supplemental Fig. S5B). We found that only 20% of PARG-alone targets are upregulated during the transition from early to late third instar larvae (Fig. 3D). PARG-alone targets are mainly involved in metabolism and cuticle formation (Supplemental Fig. S7). We identified 39 genes coding for cuticle constituents that are bound by either PARG or PARP1, with 27 being exclusively bound by PARG. Strikingly, all these PARG-bound genes exhibit a significant downregulation during the transition from larva to pupa and display a marked increase in expression in *parg* mutant individuals (Supplemental Table S4). Interestingly, we identified 12 genes involved in cuticle formation that are co-bound by both PARP1 and PARG. These genes collectively exhibit an increase of their expression levels before the larva-to-pupa transition, while they experience a significant downregulation in *parg* and *parp1* mutant animals (Supplemental Table S4). Given the well-established change in cuticle composition during the larva-to-pupa transition^26^, our findings suggest a regulatory role of PARG and PARP1 in orchestrating this transformation.

Moreover, our study identifies PARG as a key regulator of genes implicated in various metabolic functions (Supplemental Fig. S7). More precisely, we found that PARG binds to the loci of genes participating in the digestive processes (Supplemental Table S5) (and based on^27^). Strikingly, these genes exhibit a reduction in expression prior to the transition to pupa, while their expression is substantially upregulated in *parg* mutant, has we previously reported^14^. Simultaneously, our investigation unveiled PARG's independent binding to the loci of metabolic genes governing fatty acid biosynthesis, glycogen metabolism, and amino acid metabolism (Supplemental Table S6). Notably, all these metabolic genes display a downregulation before the transition to pupa, and a notable upregulation in *parg* mutant individuals. Intriguingly, we also identified four genes involved in amino acid metabolism, which are bound by both PARP1 and PARG. These genes are collectively upregulated before transition to pupa while they are downregulated in *parg* and *parp1* mutants. In summary, our results collectively demonstrate PARG’s role in repressing the expression of metabolic genes prior to the transition to the pupal stage, shedding light on its regulatory impact in these critical developmental processes.

## Discussion

As expected, we found PARP1 to be enriched at the promoter of highly active genes. Interestingly, we identified that PARG/PARP1 common targets are genes that increase their expression rapidly at the end of third instar larvae, while PARP1-alone target genes present a high expression level along all larval stages (Supplemental Fig. S5B, C). This result suggests that the presence of both PARG and PARP1 is required to increase gene expression rapidly, while only the presence of PARP1 is required to maintain a constant expression level.

**Figure 4:**
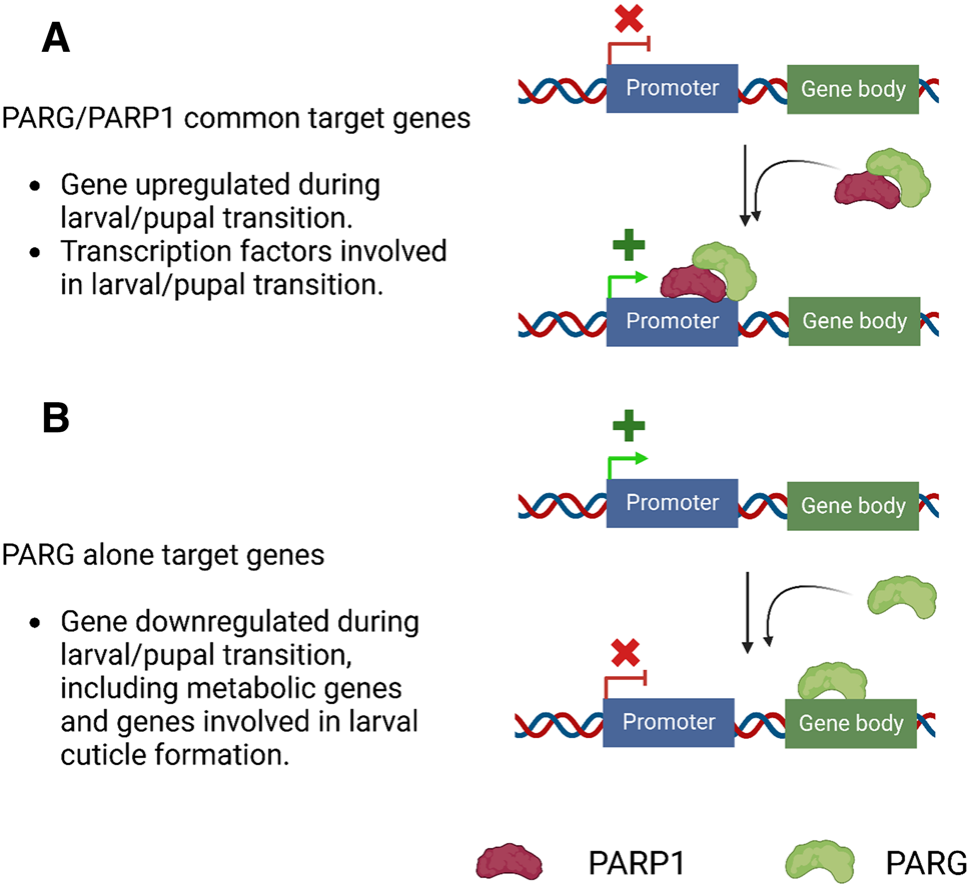
PARG exhibits two distinct mechanisms of action at chromatin. (**A**) PARG binds, together with PARP1, to the promoter region of target genes to promote their expression. PARG/PARP1 common targets are mainly genes upregulated during larval/pupal transition, including transcription factors mainly expressed at the end of the larval stage and during pupal stage. (**B**) PARG binds, without PARP1, to the gene body of its target genes to repress their expression. PARG-alone targets are mainly genes involved in metabolism and larval cuticle formation; two processes downregulated before larval/pupal transition.

PARP1 has been shown to directly regulate gene expression as an activator of transcription^28–32^, but sometimes as a repressor^18,33,34^. PARG activity is essential for PARP1 function since PARP1 mainly targets itself^18^. Accordingly, PARG was expected to participate in PARP1-mediated transcriptional regulation, at least by allowing PARP1 to return to chromatin. Despite the absence of a DBD, we showed in this study that PARG binds to chromatin, confirming the in vitro results of a previous study on human cells^35^. Remarkably, we identified two distinct PARG-binding profiles. First, PARG, together with PARP1, binds to the promoter region of genes involved in metamorphosis to promote their expression (Fig. 4A). Second, PARG binds to the gene body of genes involved in metabolism and cuticle formation to repress their expression before the transition from larval to pupal stage (Fig. 4B). Notably, it is crucial to highlight that the observed downregulation of these metabolic genes before the transition to the pupal stage does not entail complete silencing, signifying a nuanced regulatory mechanism at play. Indeed, metamorphosis is well-documented to orchestrate a U-shaped pattern in global metabolism dynamics, as corroborated by previous studies^36–39^. Specifically, metabolism rate is downregulated preceding the transition to the pupal stage and is upregulated during the middle of pupal stage.

One possibility is that *parg* mutant animals experience subtle developmental delays during the wandering stage, contributing to the observed difference between mutant and control larvae. However, this possibility is challenged by the binding of PARG to these metabolic loci, suggesting a more direct involvement of PARG in the regulation of the expression of these metabolic genes. Our findings shed light on the distinctive role of PARG, particularly in the absence of PARP1, as a key contributor to the downregulation of metabolic genes prior to pupation, simultaneously, PARG’s involvement in orchestrating changes in cuticle formation underscores its multifaceted role in shaping developmental processes. Collectively, our results offer compelling insights into the potential global transcriptional regulatory roles of both PARP1 and PARG in the context of metamorphosis, further expanding our understanding of their multifunctional contributions to this intricate biological phenomenon.

Our prior research revealed that the absence of either PARP1 or PARG leads to the upregulation of genes that are expected to be downregulated before pupation^14^. In this study, our analysis reveals that 72.1% of PARG-alone target genes exhibit upregulation in the absence of PARP1, despite the absence of direct binding by PARP1 to these genes. This outcome underscores the synergistic interplay between PARG and PARP1; the absence of one affects the function of the other^12,35^. Therefore, these genes are not directly impacted by the absence of PARP1 but by a defect in PARG function caused by the absence of PARP1. Interestingly, we found that PARG binds the loci of 4639 genes in total. This number is coherent with the range of binding sites recorded for other transcription factors that can range from 1 binding site to more than 9000 in *Drosophila*^40^. However, it is worth noting that around 30% of loci bound by PARG are not misregulated in *parg* mutant at the analyzed time-point. Collectively, the integration of our previous RNA-seq data with the current ChIP-seq results provides a more comprehensive understanding of the roles played by PARP1 and PARG in gene expression regulation before the transition from larva to pupa.

In the absence of a DNA-binding domain, it is plausible that PARG operates as part of more extensive protein complexes. When considering the shared targets of PARG and PARP1, it is reasonable to postulate that PARP1 takes on the role of recruiting PARG to the promoters of these loci. In this context, PARG effectively serves as a cofactor for PARP1. Additionally, our data concerning genes regulated by PARG indicate that PARG predominantly associates with the gene bodies rather than their promoters. This pattern of binding aligns more closely with the anticipated behavior of chromatin-associated factors.

In this study we showed that PARP1 and PARG play critical roles in the transition from larva to pupa, roles consistent with the death of both *parp1* hypomorph and *parg* null mutants before metamorphosis^11,18^. However, the expression of PARG-alone and PARG/PARP1 common targets is dynamic throughout development (Fig. 3D), not only before metamorphosis. Furthermore, we previously reported that affecting PARP1 or PARG maternal contribution leads to lethality during embryonic stage^7,19^, suggesting that PARG and PARP1 might control transcriptional changes before metamorphosis and earlier in development. We found that PARG also exhibits a chromatin-binding pattern that is PARP1-independent. PARG binds to the gene body of genes involved in cuticle formation and metabolism to repress their expression before metamorphosis. However, since PARG lacks a DBD, it is not clear how PARG is recruited to chromatin in the absence of PARP1.

The major actor involved in metamorphosis is the ecdysone pathway^24,25^. We previously reported that 20-hydroxyecdysone, an effector in the ecdysone pathway^41^, can activate PARP1 activity^12^. Furthermore, we previously showed that the expression of genes coding for enzymes involved in the synthesis of 20-hydroxyecdysone are not affected during L3 larval puff stage 7-9 in *parg* and *parp1* mutant^14^, suggesting that PARP1 acts downstream to ecdysone rather than upstream. Additionally, the expression of glue genes, which are located within puffs of polytene chromosomes and belong to the early response to ecdysone pathway that starts during L3 larval puff stage 7-9^42^, are still initiated in both *parg* and *parp1* mutants, suggesting that PARP1 does not affect ecdysone early-response genes. Interestingly, we found that PARG and PARP1 bind together to the promoter region of the transcription factors involved in ecdysone mid-response (Supplemental Table S1). Their expression is not significantly affected in *parg* and *parp1* mutants, but most are downregulated^14^. Together, these results suggest that PARP1 and PARG might play a role in the ecdysone mid-response, and that a defect in the ecdysone pathway could be the cause of the developmental arrest that exhibits both *parg* and *parp1* mutant animals. However, the broad binding of PARP1 and PARG to loci involved in the metamorphosis, infers that the function of these dual proteins is not limited to the transition from ecdysone early to mid-response but is more global in the promotion of transcription.

In conclusion, our study reveals that poly(ADP-ribosylating) enzymes directly orchestrate transcriptional changes that start before metamorphosis by promoting the expression of genes required for pupation while in parallel regulating negatively the expression of metabolic genes before the transition to pupal stage.

## Methods

### Drosophila strains and genetics

Flies were raised at 20 °C. The PARG-YFP line used in ChIP-seq corresponds to *parg*^*27.1*^*/parg*^*27.1*^*; pP{w*^*1*^*, UAST::PARG-EYFP}, 69B-GAL4/TM2*, as described in^19^. The *parg*^*27.1*^ mutant was described in^11^, *P{w*^*1*^*, UAST::PARG-EYFP}* was described in^43^, and *69B-GAL4* driver was described in^7^. The expression of PARG-EYFP rescues the lethality of *parg* mutant. The PARP1-YFP line used in ChIP-seq was *P{w*^*1*^*, UAST::PARP1-EYFP}, P{GAL4}Mz1087.hx ; parp1*^*C03256*^*/parp1*^*C03256*^. UAST-PARP1-EYFP was described in^32^ and Mz1087.hx GAL4 driver was described in^44^. The expression of PARP1-EYFP rescues the lethality of *parp1* mutant. A yellow white strain carrying the mutations *y*^*1*^*, w*^*1118*^ was used as a YFP-negative control line.

### Quantitative RT-PCR assay

This assay was performed in triplicate. Twelve wandering third instar larvae were collected for control *yellow white* and PARG-YFP flies. Total RNA was extracted from cells using the QIAshredder column and RNeasy kit (Qiagen). Contaminating genomic DNA was removed by the g-column provided in the kit. cDNA was obtained by reverse transcription using M-NLV reverse transcriptase (Invitrogen). Real-time PCR assays were run using SYBR Green master mix (Bio-Rad) and an Applied Biosystems StepOnePlusTM instrument. The amount of DNA was normalized using the difference in threshold cycle (CT) values (ΔCT) between *rpL32* and *parg* genes.

The quantitative real-time PCR (qPCR) primer sequences for Drosophila melanogaster ribosomal protein L32 gene (*rpL32*) were 5′-GCTAAGCTGTCGCAACAAAT-3′ (forward) and 5′-GAACTTCTTGAATCCGGTGGG-3′ (reverse).

Sequences for *parg* were 5′-AGAAACACCCTCAAGAGGAAG-3′ (forward) and 5′-CGCTCTGTGGGACACAC-3′ (reverse).

### Chromatin immunoprecipitation assay

ChIP was performed with PARG-YFP and PARP1-YFP lines and with a yellow white line (control line) that does not express YFP as a negative control line. We performed ChIPseq experiment on YFP-tagged PARG due to the unavailability of antibodies capable of effectively binding to *Drosophila* PARG. This experiment was performed in duplicates for PARG-YFP (two biological replicates for PARP-YFP and two biological replicates for control lines) and in triplicates for PARP1-YFP. 75 wandering third instar larvae (L3 puff stage 7-9) were collected in a 2 ml DNA LoBind Eppendorf tube and washed twice with 1 ml 1× PBS. The larvae were homogenized in ice-cold lysis buffer (200 µl of 1× protease inhibitor cocktail, 250 µl of 100 mM PMSF, 800 µl of 1× PBS, and 1 µl of Tween 20) using a pellet pestle. The homogenized lysate was crosslinked using 244.5 µl of 11% formaldehyde to a final concentration of 1.8% for 15 min at room temperature on a rotator. 500 mM Glycine was added to quench the fixative on ice for 5 min. The larval debris was pelleted at 1,000 g for 3 min, and the supernatant was removed. The pellet was resuspended in 1 ml sonication buffer (0.5% SDS, 20 mM Tris pH 8.0, 2 mM EDTA, 0.5 mM EGTA, 0.5 mM PMSF, and 1× protease inhibitor cocktail), and chromatin was fragmented to 300–500 bp by Bioruptor sonicator (UCD-200) for 20 cycles (30 s high frequency sonication, 1.5 s pause) in a cold room. The sonicated material was pelleted at 10,000 g for 10 min at 4 °C, supernatant was collected, and then fragment size was checked prior to immunoprecipitation. The sonicated chromatin was pre-cleared and incubated with an anti-GFP polyclonal antibody (TP-401, Origene) at 4 °C overnight. TP401 recognizes YFP as well. The immunoprecipitated chromatin was then collected with prewashed Protein A agarose beads for 2 h. The beads were sequentially washed with the following buffers: 1 low-salt buffer wash (0.1% SDS, 1% Triton X-100, 2 mM EDTA, 20 mM Tris442 HCL pH 8.0, and 150 mM NaCl), 3 high-salt buffer washes (0.1% SDS, 1% Triton X-100, 2 mM EDTA, 20 mM Tris–HCL pH 8.0, and 500 mM NaCl), 1 LiCL wash (2 mM EDTA, 20 mM Tris–HCl pH 8.0, and 0.25 M LiCl, 1% NP-40), and 2 TE buffer washes before elution. Bound chromatin on beads was eluted twice at room temperature using 250 µl of freshly prepared ChIP elution buffer (1% SDS and 100 mM NaHCO3) for 15 min and reverse-crosslinked overnight at 65 °C. The eluates were then treated with RNase A and proteinase K prior to DNA extraction via phenol–chloroform extraction and ethanol precipitation. The library was prepared using NEBnext multiplex oligos for Illumina kit (Index primer set 4), according to the manufacturer’s protocol. Paired-end sequencing was performed by Novogene Corporation, Sacramento CA. Raw data are available here: (GSE228898).

ChIP-qPCR assay was repeated with three biological replicates per condition for ChIP-qPCR confirmation. To confirm that PARG binds to the promoter region of *kek1* locus, we performed another set of ChIP assay on PARG-YFP and a yellow white line. This experiment was performed in triplicates. We designed two sets of primers (Supplemental Fig. S3), one set to amplify *kek1* promoter region (positive region) and one set to amplify the beginning of *kek1* gene body where PARG is not detected (negative region).

Primers used for *kek1* positive region: 5′-GTTGCGTCGTTCCCGCTGTAGC-3′ (forward) and 5′-CGGTGTGTCCTGGCTAGCGGTAC-3′ (reverse).

Primers used for *kek1* negative region: 5′-GGATCTGCATGTGGATGAGTTTGCC-3’ (forward) and 5′-GCTCGACATGTAATCGAGGCATTCTC-3′ (reverse).

Real-time PCR assays were run using SYBR Green master mix (Bio-Rad) volume and an Applied Biosystems StepOnePlus™ instrument. The amount of DNA in the immunoprecipitated fraction (IP) was compared to the Input fraction for both PARG-YFP and yellow white control lines.

### Genome-wide datasets

In this study, we compared different publicly available ChIP-seq and ATAC-seq datasets. All were performed on whole *Drosophila* third instar larvae and included PARG-YFP ChIP-seq (this study), (GSE228898). *parg* mutant RNA-seq from our previous study^14^, GSE200499. PARP1-YFP ChIP-seq, (GSE217729). *parp1* mutant microarray from our previous study^45^, ATAC-seq^22^, GSE96922. Pol II ChIP-seq^46^ (GSE15292). H3K4me3 ChIP-seq^46^, GSE49491. H3K27Ac ChIP-seq ^46^, GSE49488. H3K27me3 ChIP-seq^46^, GSE49490. H3K9me2 ChIP-seq^46^, GSE47260. H3K9me3 ChIP-seq^46^, and GSE47258. All these datasets were analyzed from raw data. In addition, we used the Developmental time-course RNA-seq dataset^23^, SRP001065.

### ChIP-seq analysis

ChIP-seq data were analyzed with Galaxy^47^. The quality of raw reads was checked using FastQC (version. 0.11.9), and adapters were removed with fastp^48^. Trimmed raw reads were aligned to the *Drosophila* genome (dm6) using Bowtie2^49^. Unmapped and low-quality reads were discarded (< = 20 mapQuality) using BamTools^50^. Duplicate reads were identified and removed from mapped reads using Picard MarkDuplicates (http://broadinstitute.github.io/picard). MACS2^51^ was used to call peaks against control (Input or negative control, depending from the dataset) using default settings. Peaks were annotated to genomic features with ChIPseeker ^52^. Pairwise correlation of peaks was determined using Intervene^53^. MACS2 bedGraph pileups were used to generate normalized coverage of ChIP-seq signals using Deeptools bigWigCompare by computing the ratio of the signals (IP *vs*. Control/Input) using a 50 bp bin size. Deeptools multiBigwigSummary and plotCorrelation^54^ were used to determine genome-wide signal correlation using a 10 kb bin size. Deeptools computematrix Deeptools plotHeatmap^54^ was used with a 50 bp bin size to create enrichment profiles around peak centers (± 1 kb) in reference mode, or in a 2 kb scale region mode from transcription start site (TSS) to transcription end site (TES) to create enrichment profiles along the genes. Scale region mode also included the 1 kb flanking regions before TSS and after TES.

Gene ontology (GO) terms were determined using g:profiler^55^. The list of *Drosophila* transcription factors was obtained from Flybase^56^ (Gene Ontology “DNA-binding transcription factor activity”). Time-course heatmap was generated with heamap2 after computing z-scores and performing an Euclidian clustering. Gene set of a specific GO-term were retrieved from AmiGO database^57,58^.

### Supplementary Information

Supplementary Information 1.

Supplementary Table S1.

Supplementary Table S2.

Supplementary Table S3.

## Data Availability

Mutant strains and transgenic stocks are available upon request. The authors state that all data necessary to confirm the conclusions presented in the article are represented fully within the article. PARG ChIP-seq raw and processed data are accessible upon demand or on GEO platform: GSE228898. PARP1 ChIP-seq raw and processed data are accessible upon demand or on GEO platform: GSE217729. The PARG ChIP-seq data can be accessed with the accession number GSE228898. Similarly, the PARP1 ChIP-seq data can be accessed with the accession number GSE217729.
