# Supplementary material for: Poly(ADP-ribosyl)ating enzymes coordinate changes in the expression of metabolic genes with developmental progression
Source: Sci Rep. 2023 Nov 20;13:20320. doi: 10.1038/s41598-023-47691-8 (PMC10661653; doi:10.1038/s41598-023-47691-8)
Supplement: Supplementary file 1 — Supplementary Information 1. [file 41598_2023_47691_MOESM1_ESM.docx]

**SUPPLEMENTARY APPENDIX FOR**

**Poly(ADP-ribosylating) enzymes coordinate metabolic pace with developmental progression.**

Guillaume Bordet^1^, Gbolahan Bamgbose^1^, and Alexei V. Tulin^1^

1 - University of North Dakota, Grand Forks, ND

**Address correspondence to:**

Email: Alexei.Tulin@und.edu

**THIS FILE INCLUDES:**

**Supplemental Figure S1.** PARG-YFP exhibits a similar expression level compared to endogenous Parg.

**Supplemental Figure S2.** PARG presents a higher occupancy at the gene body.

**Supplemental Figure S3.** PARG binds the promoter of *kek1* locus.

**Supplemental Figure S4.** Half of PARG peaks colocalized with PARP1.

**Supplemental Figure S5.** Time course expression of PARG/PARP1 common, PARG-alone and PARP1-alone target genes.

**Supplemental Figure S6.** PARG/PARP1 common target genes are involved in development and morphogenesis.

**Supplemental Figure S7.** PARG-alone target genes are involved in metabolism and cuticle formation.

**Supplemental Table S1 Legend.** List of the genes bound by either PARG or PARP1.

**Supplemental Table S2 Legend.** PARG and PARP1 bind to genes involved in metamorphosis.

**Supplemental Table S3 Legend.** List of the genes coding for transcription factors bound by both PARG and PARP1.

**Supplemental Table S4.** Genes coding for cuticle components that are bound by either PARG or PARP1.

**Supplemental Table S5.** PARG binds to genes involved in digestion.

**Supplemental Table S6.** PARG binds to metabolic genes.

**
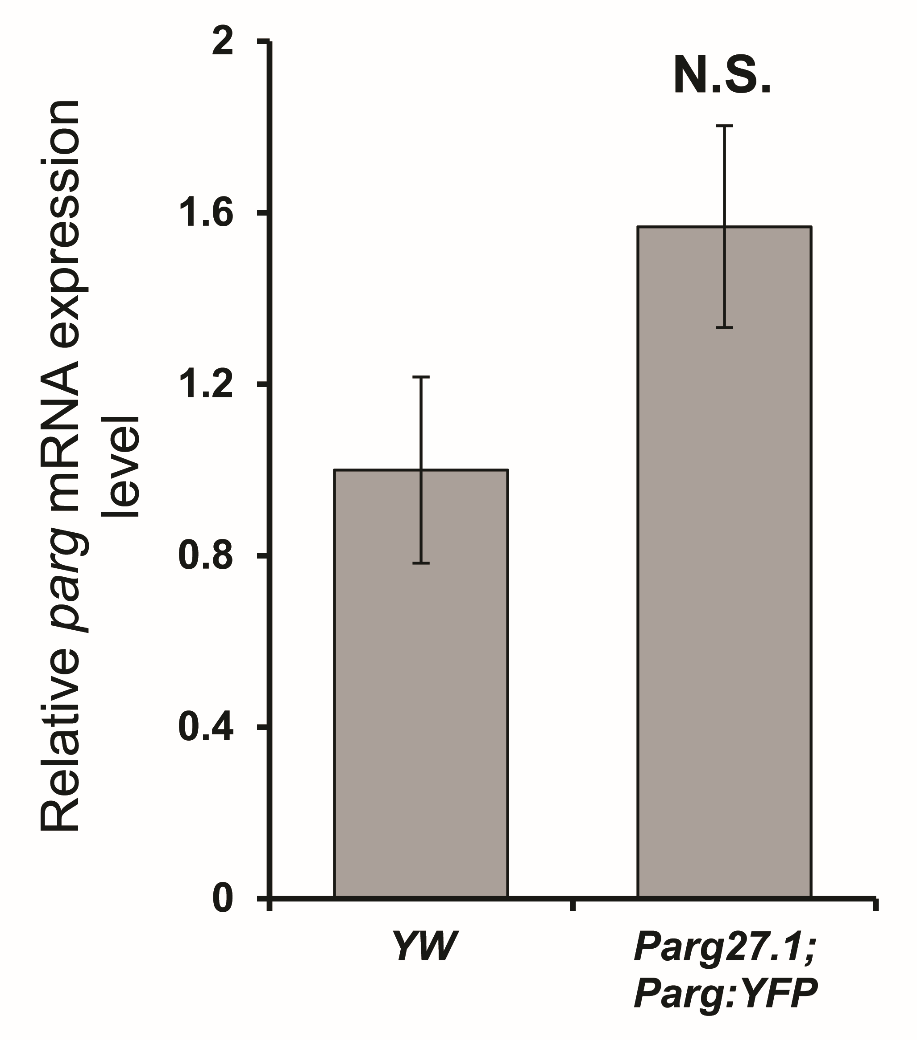
**

**Figure S1. PARG-YFP exhibits a similar expression level compared to endogenous Parg.** *Parg* mRNA expression level measured by quantitative RT-PCR for wild type flies (YW) and for PARG-YFP in endogenous *Parg* mutant background. The level is normalized to the expression level in wild type. The experiment was performed in triplicates. Error bars are Standard Error of Mean (S.E.M). The statistical test performed was a *t-*test. *p*-value= 0.0742. N.S: Non-significant.


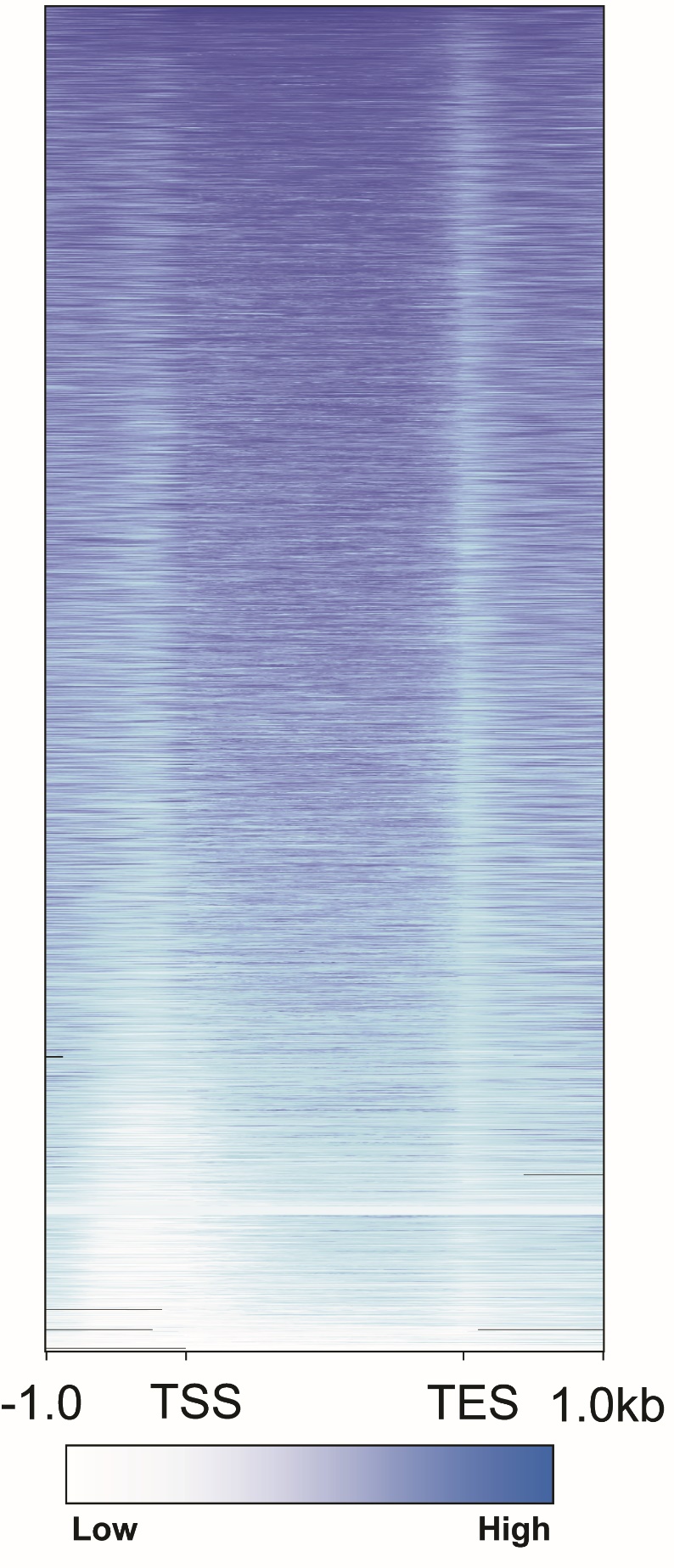


**Figure S2. PARG presents a higher occupancy at the gene body.** Heatmap showing distribution of PARG signal along PARG targets. Each line represents a gene and Lines are sorted based on intensity of PARG signal (highest at top). Regions with the lowest signal are displayed in white while regions with highest signal are displayed in dark blue. Only the genes where PARG binds to are represented.

**
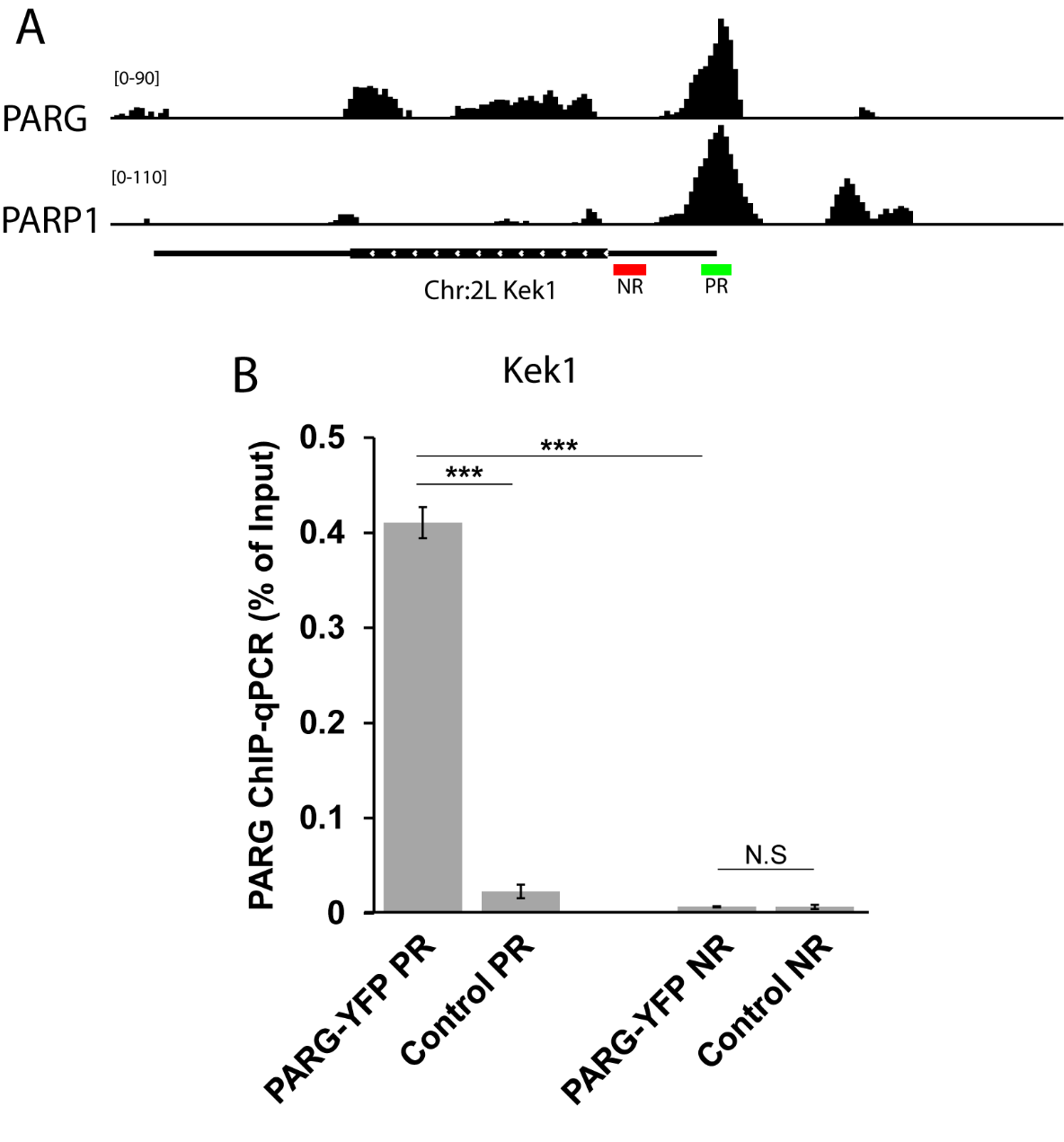
**

**Figure S3. PARG binds the promoter of *kek1* locus. A.** IGV track of Kek1 locus showing that both PARG and PARP1 bind the promoter region. PARG binding was confirmed by ChIP-qPCR, using primers inside the peak region (PR) (green) or outside, in a negative region (NR) (Red). **B.** ChIP-qPCR of Kek1 locus showing that PARG-YFP binds to the peak region (PR), but not to the negative region (NR), while control, which does not express YFP, has no binding site. Experiment was performed in triplicate. Student’s *t*-test. ***: p-value < 0.01, N.S: Non-significant.


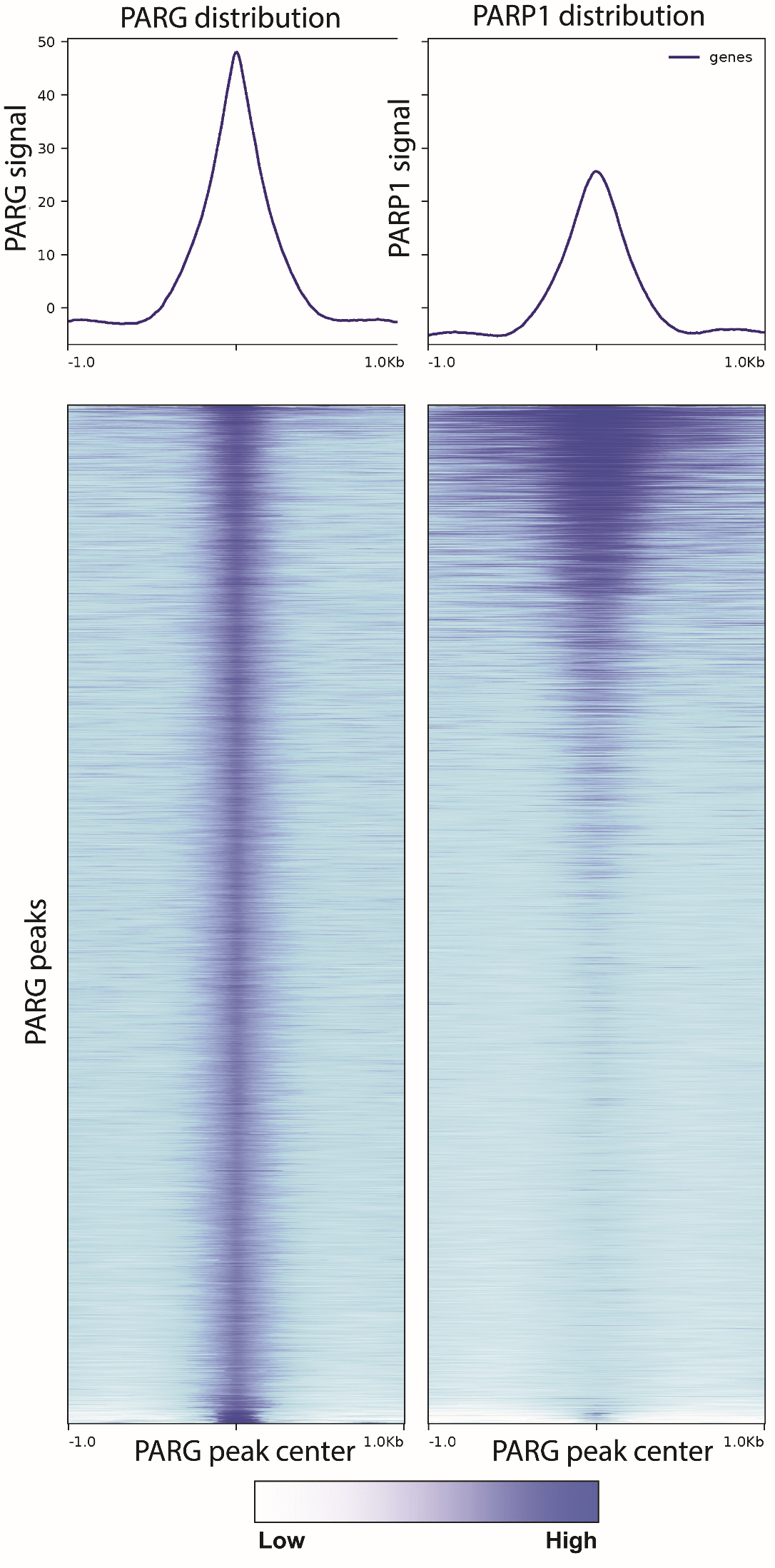


**Figure S4. Half of PARG peaks colocalized with PARP1.** Heatmap showing PARG (left) or PARP1 (right) distribution around the summit of PARG peaks. Each line corresponds to the region surrounding a single PARG peak. Peaks are sorted based on the level of PARP1 signal, highest at top. Each line displays the distribution of PARG signal (left) or PARP1 signal (right). Regions with the lowest signal are displayed in white while regions with highest signal are displayed in blue. Around half of PARG peaks colocalizes with PARP1.


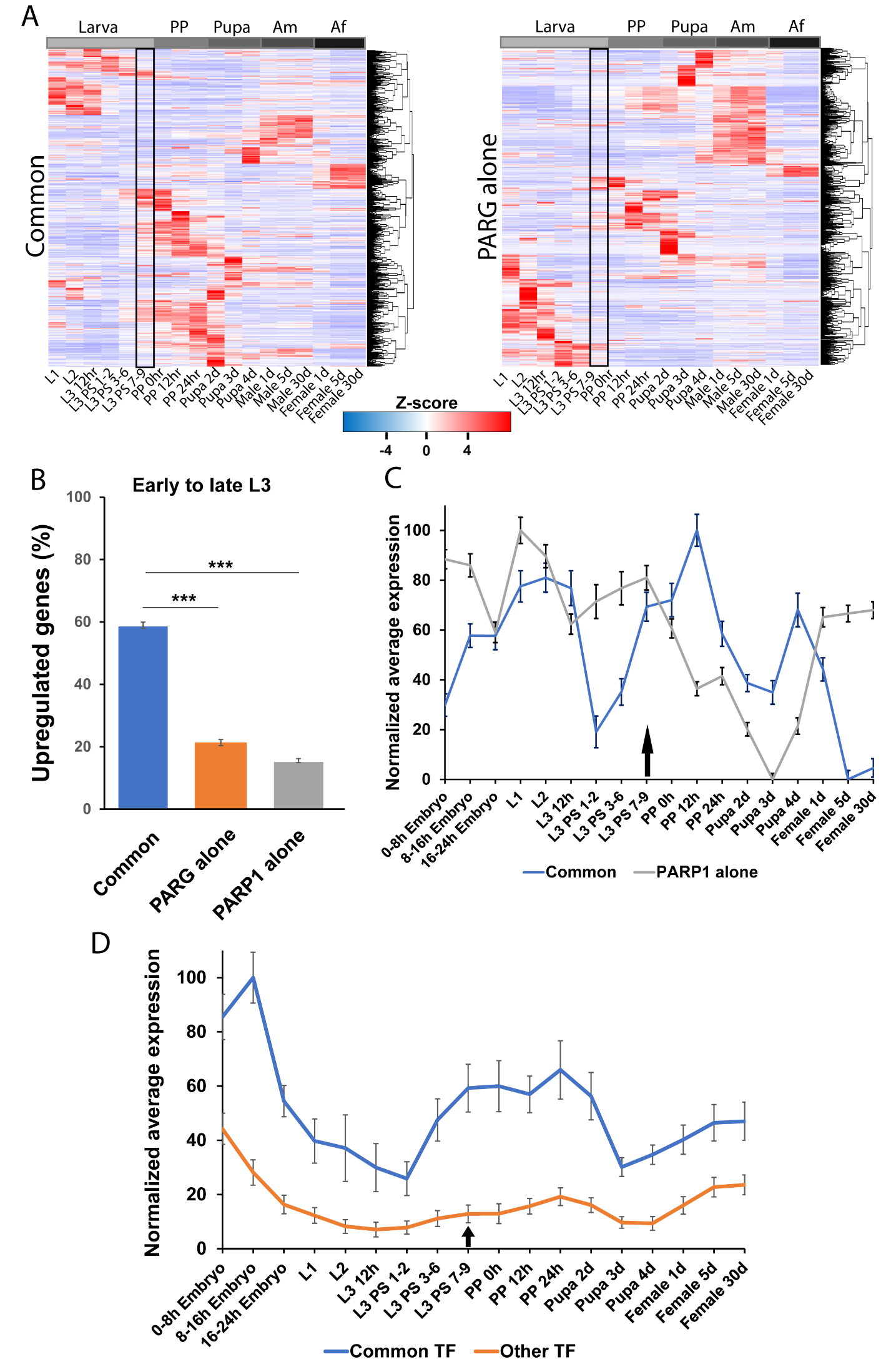


**Figure S5. Time course expression of PARG/PARP1 common, PARG-alone and PARP1- alone target genes.** **A.** Heatmap representing the time course expression of PARG/PARP1 common (left) and PARG-alone target genes (right) from first instar larvae to 30-day-old adult flies. Normalized expressions are shown as row z-scores. L1: First instar larvae, L2: Second instar larvae, L3: Third instar larvae, hr: Hours PS: puff stage, PP: Prepupa, d: Days, Am: Adult males, Af: Adult females. Time course expression data were obtained from (1). **B.** Proportion of PARG/PARP1 common (blue), PARG-alone (orange) and PARP1-alone (gray) target genes that are upregulated (fold change > 1.5) between early and late third instar larvae. Fisher’s exact test. ***: p-value < 0.01. **C.** Average time course expression of PARG/PARP1 common (blue) and PARP1-alone (gray) target genes from embryo to adult. Average expression is normalized from 0% (lowest expression) to 100% (highest expression). Error bar is standard error of the mean (SEM). Black arrow indicates the studied stage. L1: First instar larvae, L2: Second instar larvae, L3: Third instar larvae, h: Hours PS: puff stage, PP: Prepupa, d: Days. Time course expression data were obtained from (1). **D.** Average time course expression of transcription factors bound by both PARG and PARP1 (blue) and all other transcription factors (gray). Average expression is normalized to the highest expression value (100%). Error bars are Standard Error of the Mean (SEM). Black arrow indicates the studied stage. L1: First instar larvae, L2: Second instar larvae, L3: Third instar larvae, h: Hours PS: puff stage, PP: Prepupa, d: Days. Time course expression data were obtained from (1).


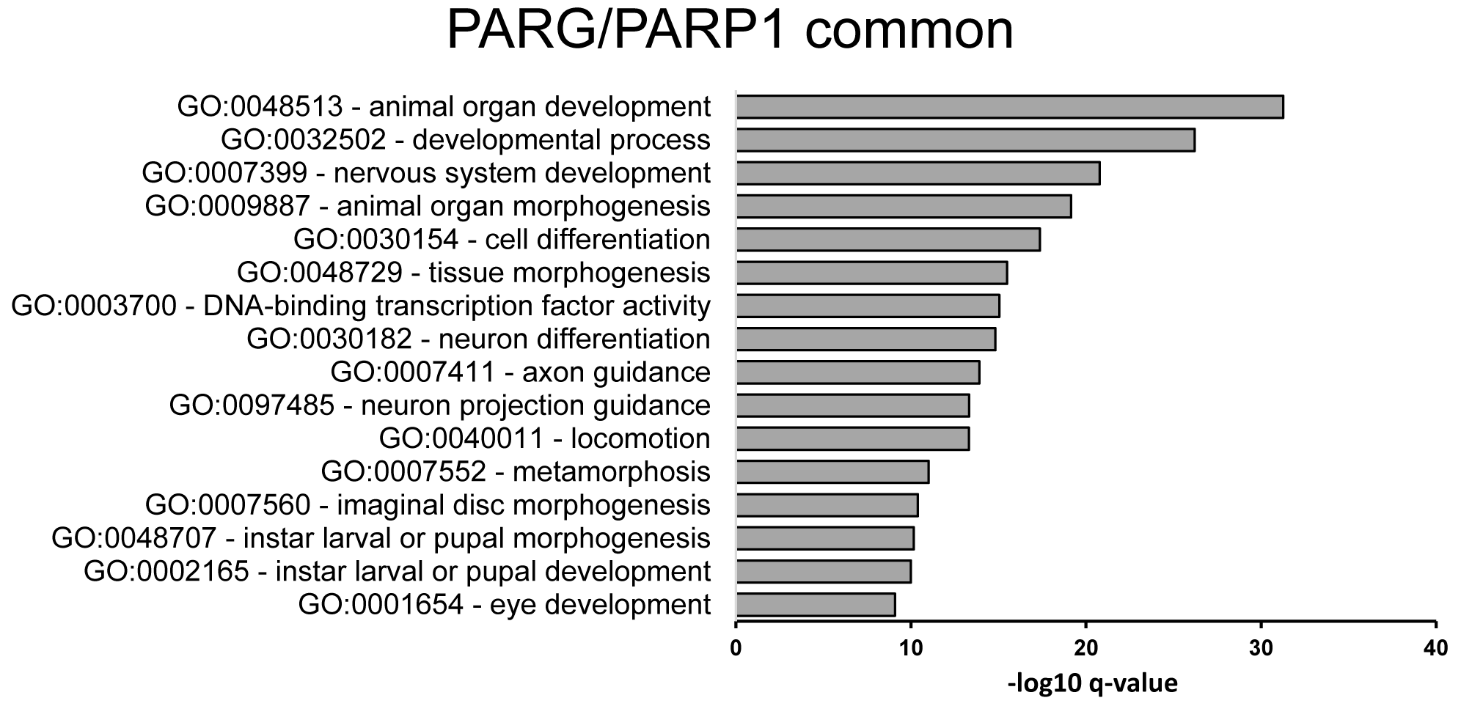


**Figure S6. PARG/PARP1 common target genes are involved in development and morphogenesis.** Gene ontology (GO) analysis of PARG/PARP1 common target genes. Only a selection of the most enriched GO-terms is presented. GO-terms for PARG/PARP1 common targets are involved in developmental, morphological, differentiation, and metamorphosis processes. q-value = False Discovery Rate (FDR)-corrected p-value. GO-terms were obtained from (2).


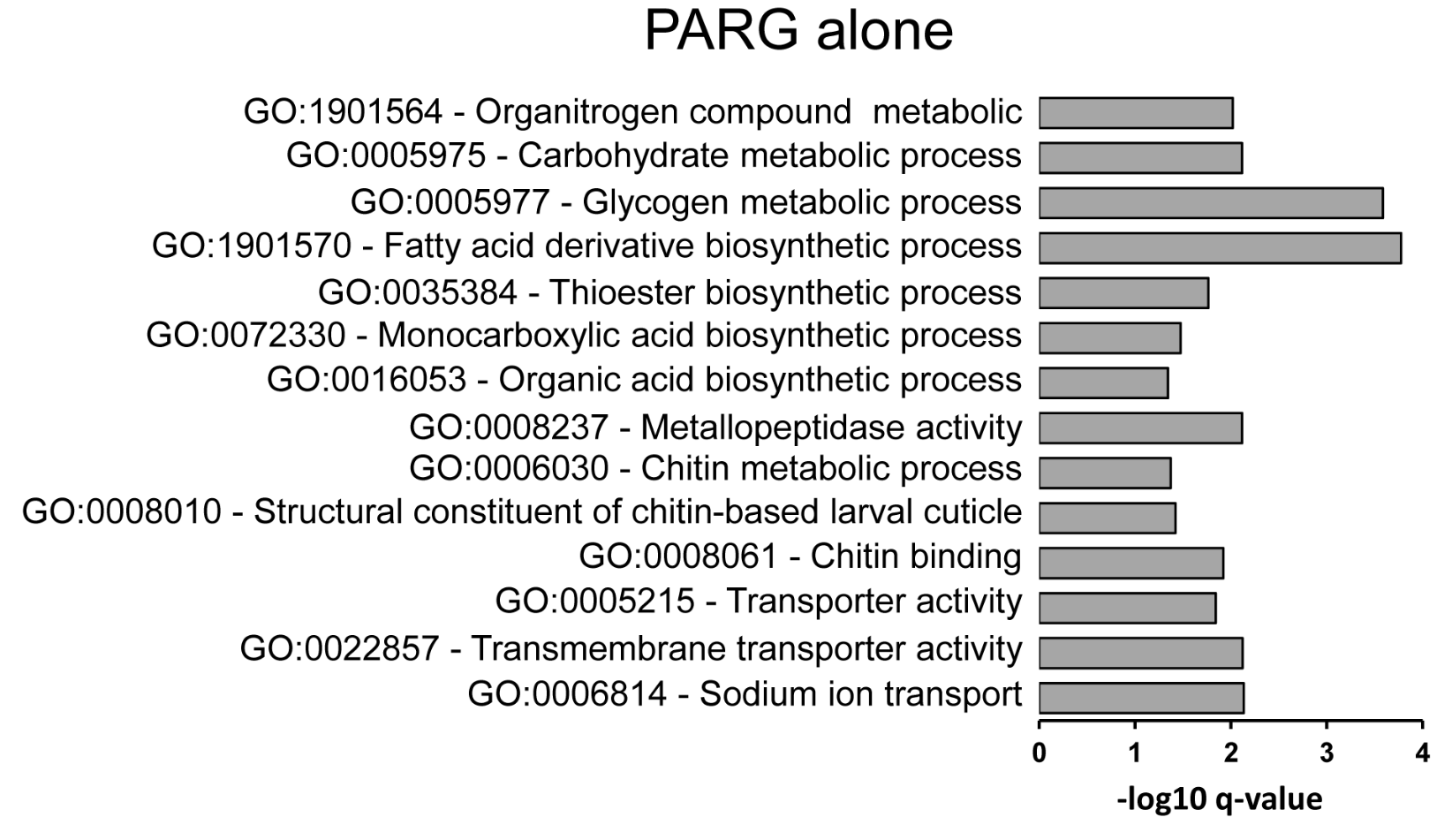
`

**Figure S7. PARG-alone target genes are involved in metabolism and cuticle formation.** Gene ontology (GO) analysis of PARG-alone target genes. Only a selection of the most enriched GO-terms is presented. GO-terms for PARG-alone target genes are involved in metabolism and cuticle formation. q-value = False Discovery Rate (FDR)-corrected p-value. GO-terms were obtained from (2).

**Supplemental Table S1 Legend.** **List of the genes bound by either PARG or PARP1.** First column: Flybase ID (FG ID). Second column: Gene name. Third column: information about if this gene is bound by PARP1 alone (PARP1), PARG along (PARG), or by both (PARG/PARP1).

**Supplemental Table S2 Legend.** **PARG and PARP1 bind to genes involved in metamorphosis.** List of the 134 genes involved in “Metamorphosis” GO-term that are bound by both PARP1 and PARG. Gene list involved in metamorphosis was retrieved from Amigo database (3, 4). 1^st^ column: Flybase ID, 2^nd^ column: Gene name, 3^rd^-10^th^ columns: Time course expression data from (retrieved from (1)). Normalized expressions are shown as row z-scores. L1: First instar larvae, L2: Second instar larvae, L3: Third instar larvae, h: Hours PS: puff stage, PP: Prepupa. 11^th^ column: PG means both PARP1 and PARG bind to this target. 12th column: Fold change of the target in a *parg* mutant context compared to control (retrieved from our previous study (5)). 13^th^ column: Fold change of the target in a *parp1* mutant context compared to control (retrieved from our previous study (6).

**Supplemental Table S3 Legend.** **List of genes coding for transcription factors bound by both PARG and PARP1.** First column: Gene name. Second Column: Log2 fold change (FC) in *parg* mutant compared to wildtype. Third column: Log2 fold change (FC) in *parp1* mutant compared to wildtype. Fourth column: type of transcription factor. Fifth column: number of transcription factors of this type bound by both PARG and PARP1 (PG) compared to the number of all transcription factors of this type (all).

| FB ID | Name | L1 | L2 | L3 12h | L3 PS1-2 | L3 PS3-6 | L3 PS7-9 | PP 0h | PP 12 h | G vs PG | PARG FC | PARP1 FC |
| --- | --- | --- | --- | --- | --- | --- | --- | --- | --- | --- | --- | --- |
| FBGN0037227 | TwdlV | -0.49 | 1.99 | 1.17 | -0.53 | -0.54 | -0.53 | -0.54 | -0.53 | G | 1.45 | 2.91 |
| FBGN0004780 | Ccp84Ad | -0.56 | 0.39 | 1.97 | -0.62 | -0.69 | -0.70 | -0.69 | 0.91 | G | 34.64 | 22.35 |
| FBGN0037225 | TwdlG | -0.38 | 2.30 | 0.57 | -0.48 | -0.50 | -0.51 | -0.52 | -0.47 | G | 12.31 | 5.18 |
| FBGN0035511 | Cpr64Ab | -0.48 | 1.85 | 1.37 | -0.56 | -0.56 | -0.56 | -0.56 | -0.50 | G | 1.74 | 1.62 |
| FBGN0035543 | CG15020 | 0.01 | 2.27 | 0.52 | -0.56 | -0.53 | -0.68 | -0.55 | -0.49 | G | 1.21 | -2.13 |
| FBGN0002440 | l(3)mbn | -0.46 | -0.69 | 1.42 | 1.70 | 0.06 | -0.60 | -0.71 | -0.72 | G | 5.33 | 5.78 |
| FBGN0004781 | Ccp84Ac | -0.48 | 2.14 | 0.92 | -0.51 | -0.52 | -0.52 | -0.52 | -0.52 | G | 31.10 | 1.43 |
| FBGN0085300 | Cpr65Ay | 0.83 | 0.30 | 0.32 | 1.82 | -0.53 | -0.85 | -0.96 | -0.94 | G | 12.87 | 30.72 |
| FBGN0033600 | Cpr47Ec | -0.56 | -0.30 | 2.33 | 0.44 | -0.49 | -0.59 | -0.59 | -0.23 | G | 68.39 | 46.16 |
| FBGN0033869 | Cpr50Cb | -0.33 | 0.57 | 2.30 | -0.48 | -0.50 | -0.53 | -0.53 | -0.50 | G | 5.51 | -3.69 |
| FBGN0037224 | TwdlF | -0.43 | 2.40 | 0.25 | -0.44 | -0.45 | -0.44 | -0.45 | -0.44 | G | 3.13 | 2.54 |
| FBGN0040950 | Muc26B | 1.92 | 0.49 | 0.59 | -0.28 | -0.06 | -0.44 | -1.08 | -1.15 | G | 2.67 | 2.48 |
| FBGN0036953 | CG17145 | 0.40 | 0.17 | -0.27 | 2.12 | 0.14 | -0.65 | -0.94 | -0.97 | G | 13.86 | 2.38 |
| FBGN0038645 | CG7714 | 1.40 | 0.73 | 0.90 | 0.08 | -0.12 | -0.37 | -1.07 | -1.55 | G | 2.80 | 1.71 |
| FBGN0034030 | CG8192 | 0.26 | 1.30 | 1.74 | -0.64 | -0.70 | -0.74 | -0.70 | -0.53 | G | 3.54 | -1.03 |
| FBGN0036952 | CG6933 | 0.87 | 0.66 | 0.14 | 0.98 | 0.62 | -0.35 | -1.40 | -1.53 | G | 2.98 | 1.55 |
| FBGN0039452 | CG14245 | 0.99 | 0.26 | 1.91 | -0.70 | -0.83 | -0.83 | -0.66 | -0.14 | G | 1.29 | 1.11 |
| FBGN0036951 | CG7017 | 1.59 | 0.45 | 0.41 | -0.03 | 0.40 | -0.08 | -1.11 | -1.64 | G | 1.93 | 1.47 |
| FBGN0260393 | CG17147 | 1.01 | 0.14 | -0.06 | 1.58 | 0.36 | -0.61 | -1.18 | -1.25 | G | 4.24 | 1.92 |
| FBGN0035607 | CG4835 | 0.47 | 0.76 | 0.11 | 1.55 | 0.31 | -0.63 | -1.26 | -1.30 | G | 12.91 | 1.90 |
| FBGN0036949 | CG7290 | 0.21 | 0.50 | -0.11 | 1.41 | 0.88 | -0.13 | -1.17 | -1.59 | G | 2.12 | 2.21 |
| FBGN0036947 | obst-F | 0.60 | 0.63 | 0.57 | 1.23 | 0.38 | -0.63 | -1.33 | -1.45 | G | 3.10 | 1.99 |
| FBGN0039454 | CG14247 | 0.64 | -0.55 | -0.18 | 1.78 | 0.77 | -0.29 | -1.22 | -0.95 | G | 3.24 | 2.72 |
| FBGN0036948 | CG7298 | 1.60 | 0.91 | 0.45 | 0.23 | -0.14 | -0.63 | -1.16 | -1.27 | G | 4.17 | 1.55 |
| FBGN0264488 | CG43896 | 0.64 | -0.56 | 0.35 | -0.03 | 1.38 | 0.64 | -0.57 | -1.87 | G | 3.29 | 1.20 |
| FBGN0038646 | CG7715 | 1.61 | 0.61 | 0.41 | 0.16 | 0.16 | -0.24 | -1.10 | -1.60 | G | 2.47 | 1.54 |
| FBGN0036220 | CG5897 | 1.34 | -0.17 | -0.51 | 0.83 | 0.44 | 0.53 | -0.63 | -1.83 | G | 2.89 | 1.97 |
| FBgn0003065 | CG5907 | -0.67 | 1.83 | 0.83 | -0.69 | -0.70 | -0.69 | -0.68 | 0.78 | PG | -3.70 | -2.09 |
| FBgn0020641 | CG5921 | -0.36 | -0.33 | -0.32 | -0.36 | -0.36 | -0.37 | -0.37 | 2.47 | PG | -2.63 | -1.85 |
| FBgn0028871 | CG5929 | -0.52 | 0.88 | 2.09 | -0.63 | -0.64 | -0.63 | -0.62 | 0.08 | PG | -1.21 | 1.30 |
| FBgn0029170 | CG5931 | -0.71 | 1.90 | 0.62 | -0.73 | -0.71 | -0.68 | -0.54 | 0.85 | PG | -4.53 | -1.83 |
| FBgn0033597 | CG5944 | -0.36 | -0.36 | -0.34 | -0.35 | -0.35 | -0.35 | -0.34 | 2.47 | PG | -2.82 | -2.64 |
| FBgn0033602 | CG5948 | -0.60 | -0.68 | -0.53 | -0.62 | -0.45 | -0.03 | 0.72 | 2.19 | PG | -3.72 | -1.31 |
| FBgn0033725 | CG5951 | -0.75 | -0.73 | -0.73 | -0.57 | 0.35 | 2.21 | 0.11 | 0.11 | PG | -1.65 | -1.42 |
| FBgn0033942 | CG5959 | -0.42 | -0.21 | -0.18 | -0.40 | -0.41 | -0.42 | -0.42 | 2.46 | PG | -1.53 | -1.75 |
| FBgn0035737 | CG5975 | -0.36 | -0.33 | -0.30 | -0.38 | -0.37 | -0.37 | -0.37 | 2.47 | PG | -1.57 | -1.87 |
| FBgn0039480 | CG6015 | -0.38 | -0.35 | -0.31 | -0.37 | -0.38 | -0.37 | -0.32 | 2.47 | PG | -1.46 | -2.10 |
| FBgn0050045 | CG6023 | -0.58 | -0.56 | -0.47 | -0.47 | -0.48 | 0.07 | 0.10 | 2.38 | PG | -2.58 | -1.55 |
| FBgn0035279 | CG5963 | -0.66 | -0.92 | 0.26 | -1.37 | 0.17 | 0.19 | 0.47 | 1.87 | PG | -1.95 | -1.39 |
|  |  | -2 |  |  |  |  |  |  |  | 2 |  |  |
|  |  |  | Z-score | | | | | | |  |  |  |

**Supplemental Table S4. Genes coding for cuticle components that are bound by either PARG or PARP1.** Genes involved in “structural constituent of cuticle” GO-term that are bound by either PARG or PARP1 (retrieved from AmiGO database (3, 4)). 1^st^ column: Flybase ID, 2^nd^ column: Gene name, 3^rd^-10^th^ columns: Time course expression data from (retrieved from (1)). Normalized expressions are shown as row z-scores. L1: First instar larvae, L2: Second instar larvae, L3: Third instar larvae, h: Hours PS: puff stage, PP: Prepupa. 11^th^ column: G means that only PARG binds to this target while PG means both PARP1 and PARG bind to this target. 12th column: Fold change of the target in a *parg* mutant context compared to control (retrieved from our previous study (5)). 13^th^ column: Fold change of the target in a *parp1* mutant context compared to control (retrieved from our previous study (6). The top panel exhibits the genes bound by PARG alone while lower panel exhibit genes bound by both PARG and PARP1.

| FB ID | Name | L1 | L2 | L3 12h | L3 PS1-2 | L3 PS3-6 | L3 PS7-9 | PP 0h | PP 12 h | G vs PG | PARG FC |
| --- | --- | --- | --- | --- | --- | --- | --- | --- | --- | --- | --- |
| FBGN0003356 | Jon99Cii | -0.50 | -0.05 | 0.60 | 1.67 | 1.00 | -0.57 | -1.03 | -1.11 | G | 3.40 |
| FBGN0003357 | Jon99Ciii | -0.44 | 0.09 | -0.14 | 2.03 | 0.76 | -0.36 | -0.87 | -1.08 | G | 2.70 |
| FBgn0019928 | Ser8 | -0.69 | -0.69 | -0.69 | 1.66 | 1.25 | 0.54 | -0.69 | -0.69 | G | 1.23 |
| FBgn0020506 | Amyrel | 1.59 | -0.53 | 1.49 | -0.50 | -0.17 | -0.07 | -0.75 | -1.06 | G | 16.30 |
| FBgn0023495 | Lip3 | 2.37 | -0.69 | -0.03 | -0.34 | -0.10 | 0.02 | -0.46 | -0.77 | G | 3.67 |
| FBgn0028945 | CG7631 | -0.30 | 1.23 | 1.24 | 1.09 | -0.71 | -0.84 | -0.85 | -0.85 | G | 35.14 |
| FBgn0028949 | CG15254 | 0.89 | 1.42 | 0.43 | 0.86 | -0.75 | -0.94 | -0.96 | -0.95 | G | 38.05 |
| FBGN0031805 | CG9505 | 0.51 | 0.03 | 0.66 | 1.25 | 0.56 | -0.33 | -0.74 | -1.95 | G | 1.27 |
| FBgn0032442 | CG15485 | 2.21 | 0.52 | -0.30 | -0.05 | -0.25 | -0.39 | -0.87 | -0.87 | G | 1.84 |
| FBgn0033296 | Mal-A7 | -0.37 | 2.35 | 0.36 | -0.36 | -0.56 | -0.55 | -0.20 | -0.66 | G | 2.55 |
| FBGN0033363 | CG13744 | -0.53 | 2.29 | 0.14 | -0.67 | -0.75 | -0.52 | -0.27 | 0.30 | G | 5.54 |
| FBgn0034052 | CG8299 | -0.67 | -0.63 | -0.43 | 2.06 | 0.96 | -0.02 | -0.59 | -0.68 | G | 3.50 |
| FBgn0035781 | CG8560 | -0.53 | -0.22 | -0.16 | 2.16 | 0.32 | 0.13 | -0.38 | -1.31 | G | 2.40 |
| FBgn0036024 | CG18180 | -0.74 | 0.05 | 0.64 | 2.02 | 0.34 | -0.58 | -0.76 | -0.97 | G | 3.59 |
| FBgn0036427 | CG4613 | -0.37 | 2.45 | 0.02 | -0.43 | -0.43 | -0.42 | -0.41 | -0.41 | G | 2.92 |
| FBgn0036738 | CG7542 | -0.63 | -0.69 | 2.28 | 0.48 | -0.48 | -0.57 | -0.39 | 0.00 | G | 1.29 |
| FBgn0036892 | Lon | 0.84 | 1.76 | 0.42 | -1.37 | -0.94 | -0.37 | -0.34 | 0.00 | G | 1.14 |
| FBGN0037230 | CG9780 | 0.46 | 0.56 | 2.12 | -0.59 | -0.65 | -0.66 | -0.67 | -0.56 | G | 5.64 |
| FBGN0037627 | CG13318 | 0.90 | 1.56 | 0.65 | 0.00 | -0.11 | -0.59 | -1.05 | -1.37 | G | 1.23 |
| FBgn0038136 | CG8774 | 1.10 | 1.12 | 1.22 | 0.06 | -0.79 | -0.90 | -0.90 | -0.91 | G | 120.05 |
| FBGN0038211 | CG9649 | 2.37 | 0.18 | 0.05 | -0.44 | -0.56 | -0.57 | -0.51 | -0.52 | G | 4.58 |
| FBGN0038507 | CG5863 | 1.68 | 1.42 | 0.13 | -0.48 | -0.58 | -0.66 | -0.76 | -0.76 | G | 4.60 |
| FBGN0038727 | CG7432 | -0.46 | 2.36 | 0.38 | -0.48 | -0.50 | -0.52 | -0.53 | -0.26 | G | 1.32 |
| FBgn0038771 | CG4390 | -2.05 | -0.13 | 0.66 | 1.03 | 0.39 | 0.62 | -0.82 | 0.30 | G | 1.02 |
| FBGN0039024 | CG4721 | 1.58 | 0.14 | 1.09 | 0.42 | -0.26 | -0.74 | -1.11 | -1.11 | G | 2.72 |
| FBGN0039252 | CG11771 | 0.47 | 1.96 | 0.82 | -0.94 | -0.83 | -0.50 | -0.46 | -0.51 | G | 1.22 |
| FBgn0039470 | CG6296 | -0.60 | 1.33 | 0.30 | 1.60 | -0.09 | -0.65 | -0.93 | -0.96 | G | 5.02 |
| FBgn0039471 | CG6295 | 0.80 | 0.63 | 1.08 | 0.78 | 0.16 | -0.83 | -1.29 | -1.34 | G | 2.44 |
| FBGN0039564 | CG5527 | 0.81 | 0.94 | 1.73 | -0.50 | -0.66 | -0.77 | -0.78 | -0.76 | G | 3.50 |
| FBGN0039609 | CG14529 | 0.54 | 0.50 | 2.10 | -0.43 | -0.70 | -0.72 | -0.69 | -0.62 | G | 9.52 |
| FBGN0039611 | CG14528 | 0.33 | 0.50 | 1.77 | 0.66 | -0.30 | -0.91 | -1.22 | -0.82 | G | 1.95 |
| FBgn0039769 | CG15534 | -0.06 | 1.70 | 0.08 | 1.32 | -0.56 | -0.81 | -0.83 | -0.83 | G | 8.42 |
| FBgn0039798 | CG11313 | -0.75 | -0.67 | -0.32 | 2.02 | 0.99 | 0.02 | -0.53 | -0.74 | G | 1.19 |
| FBgn0043576 | PGRP-SC1a | -0.26 | 2.47 | -0.38 | -0.32 | -0.33 | -0.39 | -0.40 | -0.40 | G | 1.67 |
| FBGN0050187 | CG30187 | -0.97 | -0.01 | 1.92 | 1.13 | -0.39 | -0.44 | -0.68 | -0.54 | G | 3.01 |
| FBGN0050371 | CG30371 | -0.21 | 0.54 | 1.48 | 0.82 | 0.47 | -0.54 | -1.28 | -1.29 | G | 1.79 |
| FBgn0051089 | CG31089 | 0.06 | -0.08 | 0.36 | 2.26 | -0.46 | -0.73 | -0.74 | -0.68 | G | 5.79 |
| FBgn0051198 | CG31198 | 0.50 | 1.51 | 0.40 | 1.08 | -0.61 | -0.93 | -0.96 | -0.99 | G | 10.90 |
| FBGN0051200 | CG31200 | 2.38 | -0.61 | -0.61 | -0.44 | -0.10 | -0.61 | 0.06 | -0.09 | G | 1.45 |
| FBGN0052269 | CG32269 | -0.35 | -0.35 | 2.47 | -0.35 | -0.35 | -0.35 | -0.35 | -0.35 | G | 2.25 |
| FBgn0053127 | CG33127 | 1.41 | 1.21 | 0.27 | 0.62 | -0.73 | -0.89 | -0.94 | -0.94 | G | 10.08 |
| FBGN0053128 | CG33128 | 0.56 | 0.06 | 0.14 | 1.20 | 1.08 | -0.27 | -1.12 | -1.65 | G | 2.83 |
| FBGN0250815 | Jon65Aiv | -0.50 | -0.24 | -0.20 | 2.11 | 0.76 | -0.27 | -0.54 | -1.11 | G | 3.58 |
|  |  | -2 |  |  |  |  |  |  |  | 2 |  |
|  |  |  | Z-score | | | | | | |  |  |

**Supplemental Table S5. PARG binds to genes involved in digestion.** The list of digestion enzymes were retrieved from (7). 1^st^ column: Flybase ID, 2^nd^ column: Gene name, 3^rd^-10^th^ columns: Time course expression data from (retrieved from (1)). Normalized expressions are shown as row z-scores. L1: First instar larvae, L2: Second instar larvae, L3: Third instar larvae, h: Hours PS: puff stage, PP: Prepupa. 11^th^ column: G means only PARG binds to this target. 12th column: Fold change of the target in a *parg* mutant context compared to control (retrieved from our previous study (5)).

| FB ID | Name | Gene Ontology | L1 | L2 | L3 12h | L3 PS1-2 | L3 PS3-6 | L3 PS7-9 | PP 0h | PP 12 h | G vs PG | PARG FC | PARP1 FC |
| --- | --- | --- | --- | --- | --- | --- | --- | --- | --- | --- | --- | --- | --- |
| FBgn0038731 | Acsx1R | Fatty Acid Biosynthetic Process | 0.07 | 0.58 | 2.23 | -0.47 | -0.59 | -0.60 | -0.61 | -0.61 | G | 31.87 | 7.08 |
| FBgn0037996 | CG4830 | Fatty Acid Biosynthetic Process | 1.54 | 1.11 | 0.85 | -0.26 | -0.74 | -0.82 | -0.83 | -0.83 | G | 31.09 | 15.21 |
| FBgn0038730 | Acsx1L | Fatty Acid Biosynthetic Process | 0.37 | 1.90 | 0.92 | -0.90 | -0.87 | -0.64 | -0.64 | -0.14 | G | 10.19 | 1.42 |
| FBgn0034553 | CG9993 | Fatty Acid Biosynthetic Process | 1.43 | 0.13 | -0.46 | 1.44 | 0.24 | -0.79 | -0.98 | -1.01 | G | 7.71 | 4.79 |
| FBgn0035006 | CG4563 | Fatty Acid Biosynthetic Process | 2.03 | 0.95 | 0.09 | -0.37 | -0.60 | -0.68 | -0.71 | -0.71 | G | 6.60 | 6.48 |
| FBgn0038734 | Acsx4 | Fatty Acid Biosynthetic Process | 1.23 | 1.37 | 0.18 | 0.55 | -0.30 | -0.73 | -1.25 | -1.05 | G | 3.15 | 3.52 |
| FBgn0039156 | CG6178 | Fatty Acid Biosynthetic Process | 0.98 | 0.95 | 0.69 | 1.06 | -0.73 | -1.10 | -1.12 | -0.74 | G | 2.14 | 3.20 |
| FBgn0033246 | ACC | Glycogen Metabolic Process | 0.89 | 1.21 | 1.46 | -0.52 | -0.76 | -0.79 | -0.80 | -0.70 | G | 2.63 | 4.90 |
| FBgn0283427 | FASN1 | Glycogen Metabolic Process | 0.48 | 0.96 | 1.78 | 0.01 | -0.66 | -0.88 | -0.88 | -0.82 | G | 5.65 | #N/A |
| FBgn0034143 | CG8303 | Fatty Acid Biosynthetic Process | 0.37 | 1.92 | 0.99 | -0.70 | -0.70 | -0.69 | -0.71 | -0.49 | G | 2.07 | -1.04 |
| FBgn0283427 | FASN1 | Glycogen Metabolic Process | 0.48 | 0.96 | 1.78 | 0.01 | -0.66 | -0.88 | -0.88 | -0.82 | G | 5.65 | #N/A |
| FBgn0004507 | GlyP | Glycogen Metabolic Process | 0.93 | 1.16 | 0.92 | 0.48 | -0.17 | -1.00 | -1.18 | -1.14 | G | 4.19 | 3.34 |
| FBgn0053138 | AGBE | Glycogen Metabolic Process | -0.04 | 0.34 | 2.04 | 0.64 | -0.57 | -0.93 | -0.65 | -0.83 | G | 3.97 | 3.81 |
| FBgn0265191 | Glycogenin | Glycogen Metabolic Process | -0.47 | 0.13 | 1.41 | 1.17 | 0.58 | -0.40 | -1.22 | -1.19 | G | 3.69 | 2.35 |
| FBgn0003076 | Pgm | Glycogen Metabolic Process | 0.70 | 0.50 | 1.16 | 1.07 | -0.09 | -0.90 | -1.13 | -1.30 | G | 3.28 | 4.39 |
| FBgn0033246 | ACC | Glycogen Metabolic Process | 0.89 | 1.21 | 1.46 | -0.52 | -0.76 | -0.79 | -0.80 | -0.70 | G | 2.63 | 4.90 |
| FBgn0034618 | CG9485 | Glycogen Metabolic Process | 1.02 | 0.55 | 1.02 | 0.74 | 0.13 | -0.96 | -1.17 | -1.32 | G | 2.56 | 1.34 |
| FBgn0035978 | UGP | Glycogen Metabolic Process | 0.67 | 1.00 | 1.59 | 0.04 | -0.87 | -1.23 | -0.73 | -0.46 | G | 2.06 | 1.24 |
| FBgn0036862 | Gbs-76A | Glycogen Metabolic Process | 1.07 | 0.33 | 0.05 | 0.44 | 1.20 | -0.26 | -1.26 | -1.57 | G | 1.90 | 1.61 |
| FBgn0005278 | Sam-S | SAM Biosynthetic Process | 1.83 | 0.32 | 1.12 | -0.39 | -0.55 | -0.73 | -0.80 | -0.80 | G | 2.02 | 1.72 |
| FBgn0014455 | Ahcy | SAM cycle | 1.85 | 0.51 | 1.02 | -0.58 | -0.66 | -0.70 | -0.74 | -0.70 | G | 1.36 | 1.29 |
| FBgn0034276 | Sardh | SAM cycle | 0.77 | -0.39 | 1.86 | 0.42 | -0.05 | -1.02 | -1.18 | -0.41 | G | 1.96 | 1.57 |
| FBgn0038074 | Gnmt | SAM cycle | 0.55 | 0.51 | 2.04 | -0.10 | -0.60 | -0.79 | -0.81 | -0.79 | G | 3.40 | 17.06 |
| FBgn0016013 | Faa | Tyrosine Catabolic Process | 2.03 | 0.11 | 0.96 | -0.45 | -0.65 | -0.66 | -0.67 | -0.66 | G | 5.38 | 4.15 |
| FBgn0030558 | Tat | Tyrosine Catabolic Process | 1.62 | 0.14 | 1.23 | 0.22 | -0.42 | -0.80 | -1.00 | -0.99 | G | 5.77 | 8.52 |
| FBgn0036992 | Hpd | Tyrosine Catabolic Process | 1.69 | 0.20 | 1.39 | -0.59 | -0.67 | -0.68 | -0.67 | -0.67 | G | 5.87 | 5.13 |
| FBgn0004034 | y | Melanin Biosynthetic Process from Tyrosine | -0.10 | 1.83 | 1.32 | -0.63 | -0.66 | -0.65 | -0.63 | -0.48 | G | 1.22 | 7.13 |
| FBgn0032287 | CG6415 | Glycine Catabolic Process | 0.59 | 0.34 | 1.96 | 0.17 | -0.29 | -0.82 | -0.97 | -0.98 | G | 2.46 | 2.61 |
| FBgn0037146 | CG7470 | Proline Biosynthetic Process | -0.02 | 0.27 | -0.41 | 1.02 | 1.64 | -0.08 | -1.00 | -1.42 | G | 1.43 | 1.46 |
| FBgn0037684 | CG8129 | Threonine Catabolic Process | 0.14 | 0.18 | 0.42 | 1.75 | 0.64 | -1.02 | -1.06 | -1.06 | G | 2.77 | 5.18 |
| FBgn0039052 | CG6733 | Amino Acid Metabolic Process | 1.27 | 0.05 | 1.00 | 1.00 | -0.44 | -1.11 | -1.32 | -0.47 | G | 5.51 | 7.59 |
| FBgn0039094 | CG10184 | Threonine Catabolic Process | 1.09 | 0.91 | 1.54 | -0.40 | -0.75 | -0.80 | -0.80 | -0.78 | G | 5.30 | 8.65 |
| FBgn0039153 | GatB | Glutaminyl-tRNAGln Biosynthesis via Transamidation | 0.99 | 1.64 | 0.89 | -0.79 | -0.83 | -0.67 | -0.72 | -0.51 | G | 1.40 | 1.24 |
| FBgn0039175 | beta-PheRS | Phenylalanyl-tRNA Aminoacylation | 1.26 | 1.69 | 0.42 | -0.76 | -0.69 | -0.52 | -0.53 | -0.87 | G | 1.06 | -1.14 |
| FBgn0039349 | Ssadh | Gamma-aminobutyric Acid Catabolic Process | 0.29 | 1.54 | 1.34 | 0.00 | -0.42 | -0.75 | -0.96 | -1.04 | G | 1.60 | 1.54 |
| FBgn0040211 | hgo | Tyrosine Metabolic Process | 1.57 | 0.50 | 1.35 | -0.37 | -0.73 | -0.78 | -0.78 | -0.77 | G | 21.92 | 79.71 |
| FBgn0050446 | Tdc2 | Amino Acid Metabolic Process | 2.21 | 0.72 | -0.11 | -0.56 | -0.56 | -0.56 | -0.56 | -0.56 | G | 1.23 | -1.42 |
| FBgn0051133 | Slimp | Tyrosine Catabolic Process | 0.06 | 2.00 | 0.92 | -0.08 | -0.61 | -0.74 | -0.85 | -0.71 | G | 1.34 | 1.50 |
| FBgn0005626 | ple | Tyrosine hydroxylase | -0.52 | -0.52 | -0.49 | -0.53 | -0.50 | 0.25 | 2.18 | -0.39 | PG | -4.32 | -1.84 |
| FBgn0005619 | Hdc | Histidine Catabolic Process | 1.91 | 0.11 | -0.59 | -0.76 | -0.59 | -0.03 | 2.15 | -0.30 | PG | -3.53 | -1.30 |
| FBgn0033543 | CG12338 | D-amino Acid Catabolic Process | -0.76 | -0.85 | -0.49 | 0.34 | -0.01 | 0.49 | 1.77 | -1.25 | PG | -2.31 | -1.04 |
| FBgn0014427 | CG11899 | L-serine Biosynthetic Process | -1.03 | -0.65 | -0.85 | -1.09 | -0.48 | 0.62 | 1.41 | 1.03 | PG | -2.16 | -2.26 |
|  |  |  | -2 |  |  |  |  |  |  |  | 2 |  |  |
|  |  |  |  | Z-score | | | | | | |  |  |  |

**Supplemental Table S6. PARG binds to metabolic genes.** Examples of metabolic genes that are bound by PARG. 1^st^ column: Flybase ID, 2^nd^ column: Gene name, 3^rd^-10^th^ columns: Time course expression data from (retrieved from (1)). Normalized expressions are shown as row z-scores. L1: First instar larvae, L2: Second instar larvae, L3: Third instar larvae, h: Hours PS: puff stage, PP: Prepupa. 11^th^ column: G means only PARG binds to the target while PG means both PARP1 and PARG bind to this target. 12th column: Fold change of the target in a *parg* mutant context compared to control (retrieved from our previous study (5)). Top panel display genes belonging to the “Fatty acid biosynthetic process” GO-term, middle panel the genes belonging to the “Glycogen metabolic process” GO-term, and lower panel the genes belonging to the “Amino acid metabolic process” GO-term. The gene sets belonging to these GO-terms were retrieved from AmiGO database (3, 4).

**References**

1. Graveley BR, Brooks AN, Carlson JW, Duff MO, Landolin JM, Yang L, et al. The developmental transcriptome of Drosophila melanogaster. Nature. 2011;471(7339):473-9.

2. Raudvere U, Kolberg L, Kuzmin I, Arak T, Adler P, Peterson H, et al. g:Profiler: a web server for functional enrichment analysis and conversions of gene lists (2019 update). Nucleic Acids Res. 2019;47(W1):W191-W8.

3. Ashburner M, Golic KG, Hawley RS. Drosophila: a laboratory handbook: Cold spring harbor laboratory press; 2004.

4. Gene Ontology C, Aleksander SA, Balhoff J, Carbon S, Cherry JM, Drabkin HJ, et al. The Gene Ontology knowledgebase in 2023. Genetics. 2023;224(1).

5. Bordet G, Karpova I, Tulin AV. Poly(ADP-ribosyl)ating enzymes cooperate to coordinate development. Sci Rep. 2022;12(1):22120.

6. Bordet G, Lodhi N, Guo D, Kossenkov A, Tulin AV. Poly(ADP-ribose) polymerase 1 in genome-wide expression control in Drosophila. Sci Rep. 2020;10(1):21151.

7. Lemaitre B, Miguel-Aliaga I. The digestive tract of Drosophila melanogaster. Annu Rev Genet. 2013;47:377-404.
